# Source-to-tap investigation of the occurrence of nontuberculous mycobacteria in a full-scale chloraminated drinking water system

**DOI:** 10.1128/aem.00609-24

**Published:** 2024-08-07

**Authors:** Katherine S. Dowdell, Sarah C. Potgieter, Kirk Olsen, Soojung Lee, Matthew Vedrin, Lindsay J. Caverly, John J. LiPuma, Lutgarde Raskin

**Affiliations:** 1 Department of Civil and Environmental Engineering, University of Michigan, Ann Arbor, Michigan, USA; 2 Department of Pediatrics, University of Michigan Medical School, Ann Arbor, Michigan, USA; Centers for Disease Control and Prevention, Atlanta, Georgia, USA

**Keywords:** nontuberculous mycobacteria, drinking water, opportunistic pathogens, *Mycobacterium*, building plumbing

## Abstract

Nontuberculous mycobacteria (NTM) in drinking water are a significant public health concern. However, an incomplete understanding of the factors that influence the occurrence of NTM in drinking water limits our ability to characterize risk and prevent infection. This study sought to evaluate the influence of season and water treatment, distribution, and stagnation on NTM in drinking water. Samples were collected source-to-tap in a full-scale, chloraminated drinking water system approximately monthly from December 2019 to November 2020. NTM were characterized using culture-dependent (plate culture with matrix-assisted laser desorption ionization-time-of-flight mass spectrometry [MALDI-TOF MS] isolate analysis) and culture-independent methods (quantitative PCR and genome-resolved metagenomics). Sampling locations included source waters, three locations within the treatment plant, and five buildings receiving water from the distribution system. Building plumbing samples consisted of first draw, 5-min flush, and full flush cold-water samples. As the study took place during the COVID-19 pandemic, the influence of reduced water usage in three of the five buildings was also investigated. The highest NTM densities source-to-tap were found in the summer first draw building water samples (10^7^ gene copies/L), which also had the lowest monochloramine concentrations. Flushing was found to be effective for reducing NTM and restoring disinfectant residuals, though flush times necessary to improve water quality varied by building. Clinically relevant NTM species, including *Mycobacterium avium*, were recovered via plate culture, with increased occurrence observed in buildings with higher water age. Four of five NTM metagenome-assembled genomes were identified to the species level and matched identified isolates.

**IMPORTANCE:**

NTM infections are increasing in prevalence, difficult to treat, and associated with high morbidity and mortality rates. Our lack of understanding of the factors that influence NTM occurrence in drinking water limits our ability to prevent infections, accurately characterize risk, and focus remediation efforts. In this study, we comprehensively evaluated NTM in a full-scale drinking water system, showing that various steps in treatment and distribution influence NTM presence. Stagnant building water contained the highest NTM densities source-to-tap and was associated with low disinfectant residuals. We illustrated the differences in NTM detection and characterization obtained from culture-based and culture-independent methods, highlighting the complementarity between these approaches. We demonstrated that focusing NTM mitigation efforts in building plumbing systems, which have the highest NTM densities source-to-tap, has potential for immediate positive effects. We also identified steps during treatment that increase NTM levels, which provides beneficial information for utilities seeking to reduce NTM in finished water.

## INTRODUCTION

Nontuberculous mycobacteria (NTM) respiratory infections are a significant and growing health concern, with numerous recent studies reporting increasing prevalence (1–3). The impact of NTM infections is illustrated in high associated morbidity, mortality, and healthcare costs compared to infections by enteric waterborne pathogens (4–7). Though the sources of NTM infections are often difficult to determine, exposure to drinking water populations is believed to be a major route of infection (8, 9). However, the factors that shape NTM types and levels at the tap are poorly understood. This limitation is due to several reasons, including the complexity of quantification methods, the lack of regulations governing NTM in drinking water, and the difficulty in linking specific sources to infections (10). A greater understanding of NTM occurrence, species composition of NTM, and factors that contribute to higher NTM levels in drinking water environments is crucial for the development of quantitative risk assessments and risk mitigation strategies (11, 12).

Building plumbing characteristics have been shown to greatly influence tap water quality. Building plumbing properties, including high surface-to-volume ratios, periods of stagnation, and equilibration with building temperatures, create an environment that contributes to the growth of biofilms and high densities of certain opportunistic human pathogens (OPs), including NTM (13, 14). Although previous studies have found that building plumbing may contribute to high densities of NTM, few studies have evaluated the impacts of flushing, seasonal variations, and water quality parameters (15–18). Previous studies have found densities of NTM in building plumbing as high as 10^5^–10^7^ gene copies per liter (16, 19–21). The distribution system has also been shown to influence NTM, with higher densities of NTM or greater occurrence of clinically relevant species linked to higher distribution system residence times (15, 22). Source water type (23) and drinking water treatment processes may also influence the NTM entering the distribution system, though few studies have investigated these topics in full-scale systems (24–28). Another factor linked to NTM occurrence is the type of secondary disinfectant used, with several studies reporting increased rates of NTM detection or higher NTM densities in distribution systems using monochloramine (23, 29–31). The COVID-19 pandemic brought additional concerns about OPs in building water due to low water use (32). Although most studies focused only on *Legionella pneumophila* (33–35), one study reported *Mycobacterium avium* complex densities up to 10^5^ gene copies per liter in stagnant building plumbing water (17).

Culture-based methods have long been the primary means for detecting NTM in environmental samples. Culture-based methods are often favored because they detect viable bacteria that can be used for additional analyses. However, existing culture-based methods for recovery of NTM from drinking water lack standardization, are laborious, and, despite the use of decontamination or antibiotic-containing media, are often limited in their specificity for NTM, meaning that additional methods are required to confirm species identity (36–38). Furthermore, species- and strain-level variation in susceptibility to decontamination treatments and antibiotics may introduce bias in quantifying NTM populations in mixed communities in environmental samples (37–39). Quantitative PCR (qPCR) is often favored by researchers for quantifying NTM because it is rapid and typically highly specific at the genus or species level (40–44). However, unless viability pre-treatments are used (e.g., propidium monoazide or ethidium monoazide) (45–47), qPCR does not provide information regarding viability. Recently, amplicon sequencing and shotgun metagenomics have been used to determine the relative abundance of NTM in microbial communities, with some studies reporting the enrichment of NTM in building plumbing biofilms (15, 48–50). However, DNA sequencing methods may fail to detect low-abundance microorganisms like NTM, they do not show viability, and the PCR employed in amplicon sequencing introduces bias (51, 52). Additionally, sequencing methods can only yield relative, rather than absolute abundances unless quantitative metagenomics is employed (53, 54). NTM are also difficult to lyse, which can impact DNA recovery and NTM detection (55). As methods for NTM quantification are not standardized, cross-study comparisons are challenging, particularly between studies that use culture-based methods vs those that use culture-independent methods.

The risk to human health associated with NTM in drinking water is also dependent on the species present. Species within *Mycobacterium* range from non-pathogenic, to OPs (e.g., *M. avium* and *Mycobacterium abscessus*)*,* to non-environmental pathogens (e.g., *Mycobacterium tuberculosis*). Although the risk of infection due to OPs is highly dependent on the susceptibility of the host, it is estimated that approximately one dozen of the nearly 200 species of NTM are capable of causing human infection (56). Additionally, regional differences have been observed regarding which NTM species are most likely to cause human infection. Such regional differences could result in underestimating risk if the most appropriate species are not targeted (57, 58). Due to the difficulties in identifying NTM to the species or complex level, drinking water studies often quantify total NTM, with many targeting the ATP synthase subunit c (*atpE*) gene (16, 59–61), or only investigating one or a few species of concern, such as *M. avium* or *Mycobacterium intracellulare* (20, 23, 62, 63). Identification of NTM to the species level in colonies from plate culture is typically done using PCR and Sanger sequencing targeting genes such as β-subunit of RNA polymerase (*rpoB*) or heat shock protein 65 (*hsp65*) (64–67). Another alternative for colony identification is matrix-assisted laser desorption ionization-time-of-flight mass spectrometry (MALDI-TOF MS), which is a method that analyzes proteins and generates spectra that are matched to a spectral database (68, 69). Advantages of MALDI-TOF MS include that it is rapid, accurate, and commonly used in clinical laboratories (70, 71). However, one disadvantage of MALDI-TOF MS is that misidentifications occasionally occur at the species or complex level for closely related species, including for certain clinically relevant NTM, such as *M. abscessus* and *Mycobacterium chelonae* (72). Culture-independent methods for species-level identification of NTM typically employ short-read or long-read amplicon sequencing, with targets such as the *rpoB* or *hsp65* gene (15, 48, 73).

Although previous studies have quantified NTM at various locations in drinking water systems (15, 16, 29, 31, 74), few have conducted full, source-to-tap assessments (75). This study sought to characterize the factors that shape NTM at various stages of treatment and distribution, including impacts of source water selection, individual treatment processes [e.g., ozonation, granular activated carbon (GAC) biofiltration, and disinfection], distribution, and stagnation in building plumbing. To this end, samples were collected approximately monthly over 1 year in a full-scale chloraminated system. Samples were collected from source waters (river and well water), through the drinking water treatment plant (WTP), and in five buildings (Sites A-E) served by the WTP. The estimated distribution system water ages for the five buildings ranged from approximately 16 hours at Site A to approximately 68 hours at Site E (Table S1). Building plumbing samples included first draw, 5-min flush, and full flush cold water samples. Samples were analyzed for routine physicochemical parameters, heterotrophic plate counts (HPC), and the presence of NTM, which were characterized using plate culture with MALDI-TOF MS for colony identification, qPCR, and genome-resolved metagenomics. The onset of the COVID-19 pandemic and associated building closures occurred during the study, impacting water use at three of the buildings (Sites A, B, and E), facilitating an investigation of the impact of extended low water use on NTM and building water quality. The influence of stagnation was investigated through the collection of samples from buildings with various levels of flushing.

## RESULTS

### NTM plate culture and MALDI-TOF MS

Presumptive NTM plate culture results ranged from less than the lower limit of detection (LLOD) of 1 colony-forming unit (CFU) per volume (no colonies) to above the upper limit of detection (ULOD) of 300 CFU per volume (too numerous to count [TNTC], Fig. S1; Table S2). The highest plate culture result within the quantification range was 7.3 × 10^4^ CFU/L. LLOD values ranged from 1 to 333 CFU/L and varied based on the sample volume (Table S3). Median results in the source waters and through the WTP ranged from 18 CFU/L in the ozone effluent (*n* = 6) to 45 CFU/L in the filter effluent (*n* = 6). In the finished water, the median was 4 CFU/L (*n* = 8). The highest results occurred in the first draw samples at Sites A (median: >1.2 × 10^4^ CFU/L, *n* = 7) and B (median: >1.2 × 10^4^ CFU/L, *n* = 7). The median first draw results in the other buildings ranged from 52 CFU/L at Site E (*n* = 7) to 62 CFU/L at Site C (*n* = 7). Five-minute flush and full flush sample median results ranged from 8 CFU/L in the Site E 5-min flush samples (*n* = 7) to 2.4 × 10^2^ CFU/L in the Site B 5-min flush (*n* = 7).

A subset of the colonies from the NTM culture plates (*n* = 322), representing 47 of the samples, were identified using MALDI-TOF MS. Of these isolates, 60% (*n* = 194) were identified as *Mycobacterium*, 34% (*n* = 108) were not identified, indicating that they were other bacterial or fungal species not represented in the available spectral databases, and 6% (*n* = 20) were identified as other genera including *Bacillus*, *Paenibacillus*, *Brevibacillus*, and *Micromonospora*, which all consist of endospore-forming bacterial species (Table 1; Table S4) (76–79). Within the isolates identified as NTM, 10 species or groups were identified, including *Mycobacterium arupense*, *Mycobacterium asiaticum*, *Mycobacterium aurum*, *M. avium*, *M. chelonae* complex, *Mycobacterium franklinii*, *Mycobacterium gordonae*, *Mycobacterium llatzerense*, *Mycobacterium mucogenicum/phocaicum* group, and *Mycobacterium peregrinum*. Of the recovered NTM species, *M. avium* and *M. chelonae* complex are of particular clinical relevance based on the prevalence of infections (56, 80, 81).

**Fig 1:**
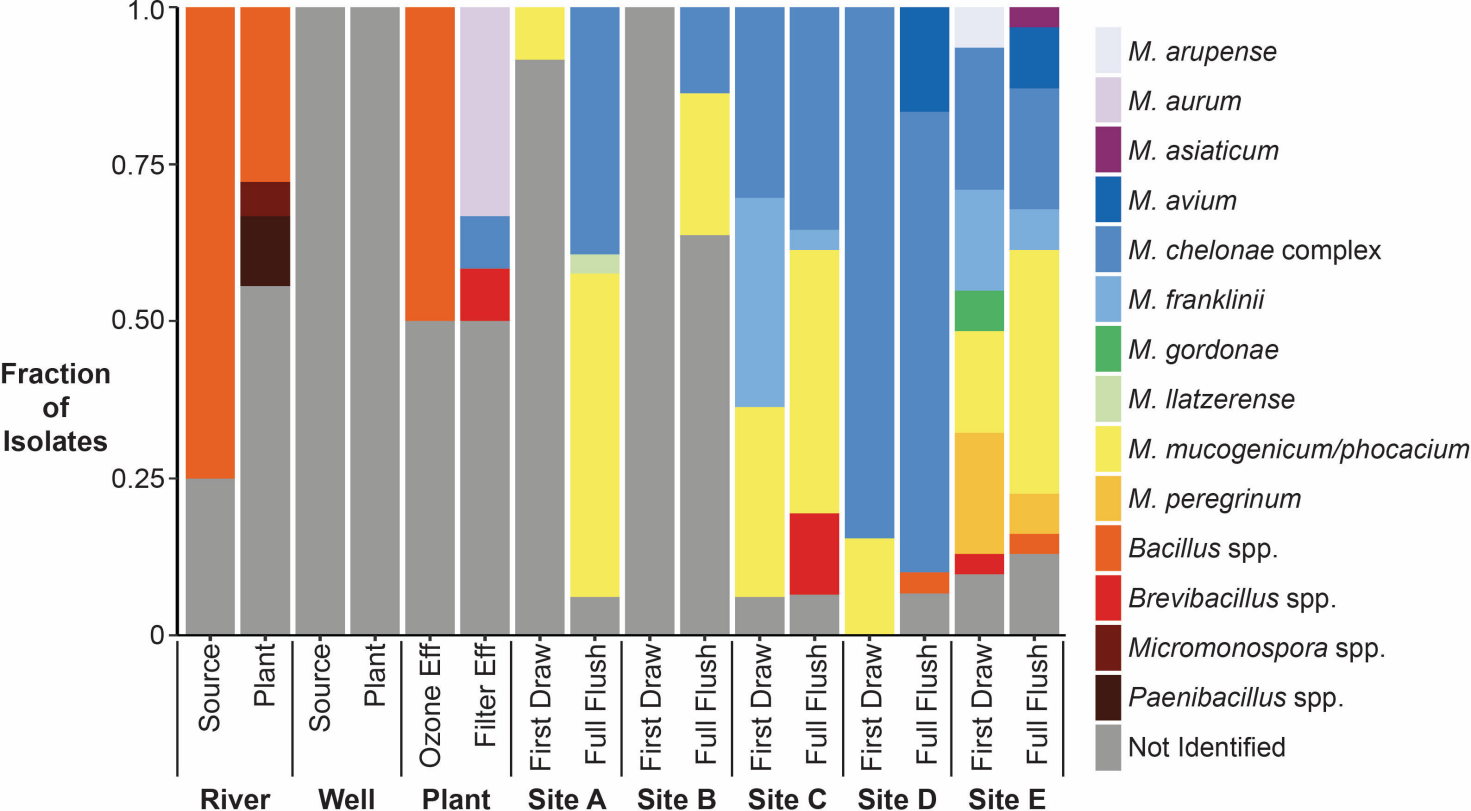
Results of matrix-assisted laser desorption ionization-time-of-flight mass spectrometry (MALDI-TOF MS) analysis for isolates from the NTM culture plates. No finished water isolates were recovered for the months analyzed. Not Identified: Sample spectra did not match spectral databases.

MALDI-TOF MS results showed that none of the isolates recovered from the source waters and WTP ozone effluent and selected for identification were *Mycobacterium* spp. (Fig. 1). In the river samples, River-Source isolates included three *Bacillus* spp. isolates and one isolate that could not be identified. For the River-Plant isolates, a larger fraction of isolates could not be identified (55%, *n* = 10), and the remaining isolates were identified as *Bacillus* spp. (28%, *n* = 5), *Paenibacillus* spp. (11%, *n* = 2), and *Micromonospora* sp. (6%, *n* = 1). All isolates from the well samples (Well-Source and Well-Plant) were microorganisms that could not be identified (*n* = 46). Of the two isolates from the ozone effluent samples, one could not be identified, and the other was identified as a *Bacillus* sp.

**TABLE 1:** Results of matrix-assisted laser desorption ionization–time-of-flight mass spectrometry (MALDI-TOF MS) analysis of the plate culture isolates

Category	Identification	Number of isolates (% of total)	Score range (median)
*Mycobacterium*	*Mycobacterium arupense*	2 (<1%)	1.92–1.96
	*Mycobacterium asiaticum*	1 (<1%)	1.61
	*Mycobacterium aurum*	4 (1%)	1.98–2.27 (2.13)
	*Mycobacterium avium*	8 (2%)	1.99–2.20 (2.09)
	*Mycobacterium chelonae* complex	84 (26%)	1.62–2.12 (1.88)
	*Mycobacterium franklinii*	19 (6%)	1.60–1.98 (1.77)
	*Mycobacterium gordonae*	2 (<1%)	1.81–2.01
	*Mycobacterium llatzerense*	1 (<1%)	1.84
	*Mycobacterium mucogenicum*/*phocaicum* group	65 (20%)	1.82–2.39 (2.11)
	*Mycobacterium peregrinum*	8 (2%)	2.10–2.31 (2.27)
Non-NTM	*Bacillus* spp.	11 (3%)	1.70–2.34 (1.94)
	*Brevibacillus* spp.	6 (2%)	1.77–2.40 (1.96)
	*Micromonospora* sp.	1 (<1%)	1.73
	*Paenibacillus* spp.	2 (<1%)	1.82–2.00
Not identified	–^*a*^	108 (34%)	0.93–1.68 (1.21)

^*a*^ –, no entry.

NTM isolates were identified in the WTP filter effluent and all downstream sampling locations, except the first draw samples from Site B. In the filter effluent, isolates included *M. aurum* (*n* = 4), *M. chelonae* complex (*n* = 1), *Brevibacillus* sp. (*n* = 1), and six isolates that could not be identified. No isolates from the finished water were analyzed using MALDI-TOF MS due to the low number of isolates recovered. In the distribution system, the majority of isolates (79%, *n* = 189 of 240) were NTM, with *M. chelonae* complex (*n* = 83) and *M. mucogenicum/phocaicum* group (*n* = 65) being the most frequently recovered. At Sites A and B, the fractions of NTM isolates recovered from first draw samples were low (<10%) but increased in the full flush samples (>35%). Increased recovery and diversity of NTM were observed at higher water age sites. At Sites D and E, the fractions of isolates identified as NTM were 93% (*n* = 40 of 43) and 85% (*n* = 53 of 62), respectively. The highest diversity in NTM isolates was observed at Site E, where eight different NTM species or groups were identified (*M. arupense*, *M. asiaticum, M. avium*, *M. chelonae* complex, *M. franklinii*, *M. gordonae*, *M. mucogenicum/phocaicum* group, and *M. peregrinum*). *M. avium* was only isolated from the full flush samples from Site D (*n* = 5) and Site E (*n* = 3).

The fraction of isolates identified as NTM for each sample analyzed using MALDI-TOF MS was used to adjust the presumptive NTM plate culture results, yielding the adjusted plate counts (Fig. S2). Although the highest presumptive CFU/L results occurred in the first draw samples from Sites A and B, adjusted NTM CFU/L results were lower due to the large fraction of non-NTM isolates. The sample with the highest adjusted NTM plate count was the July Site C first draw sample at 5.6 × 10^2^ CFU/L. As most isolates picked from the Site C, D, and E culture plates were identified as NTM, the adjusted plate counts were generally similar to the presumptive NTM counts.

### NTM quantification using qPCR

**Fig 2:**
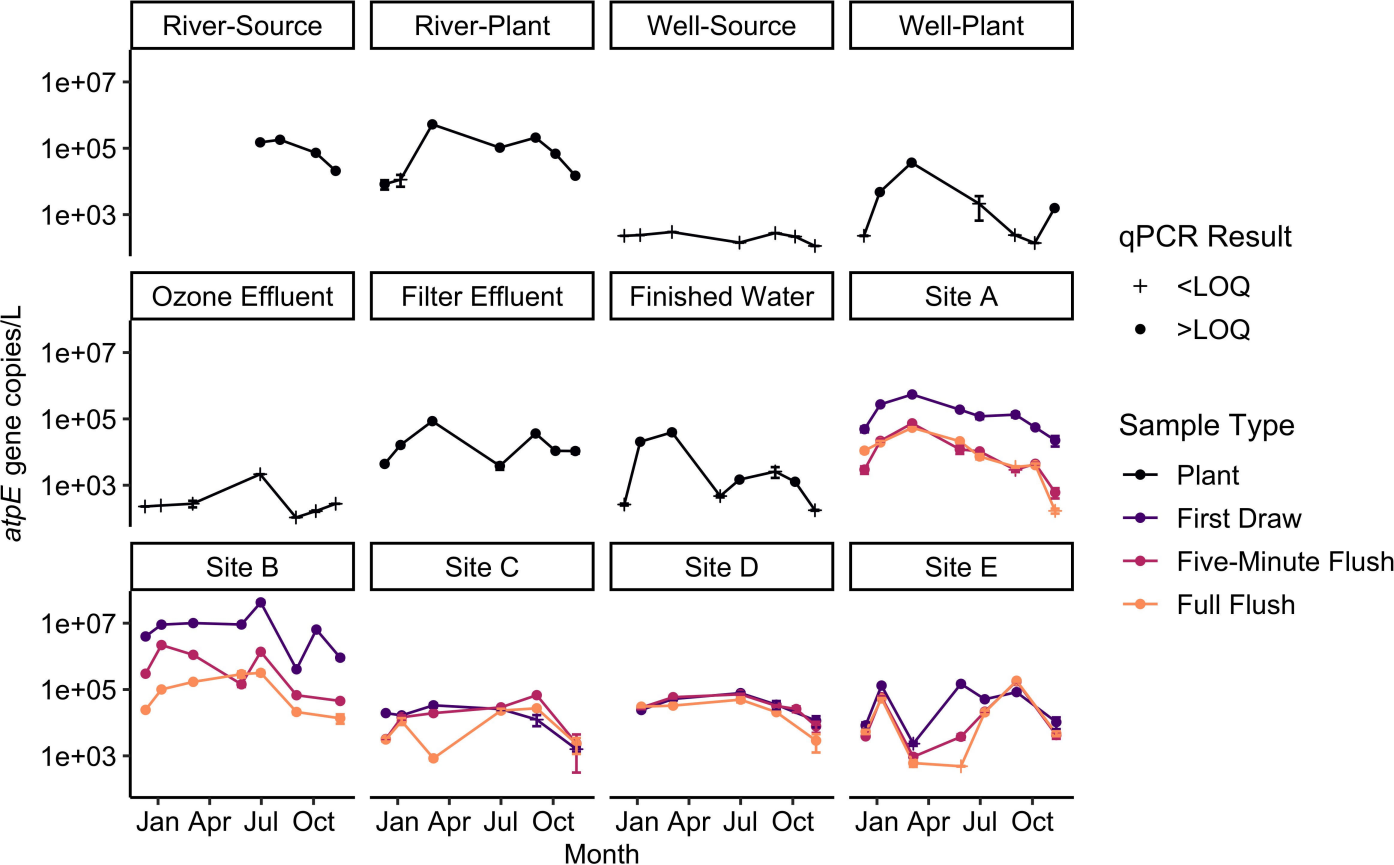
NTM *atpE* gene copy densities in all samples analyzed over the study period. Values below the LOQ (crosses) were set to one-half the LOQ and then converted to gene copies per liter. Values above the LOQ are shown as circles. Error bars show one standard deviation above and below the mean of triplicate qPCR reactions. Based on data from Dowdell et al. (44).

Similar to the plate culture results, the highest NTM densities using qPCR were observed in the first draw samples from Sites A and B (Fig. 2; Table S5). The lowest NTM gene copy densities were found in the Well-Source and the Ozone Effluent, where all samples were below the limit of quantification (LOQ) of 41 gene copies per reaction (gc/rxn). LOQs in gene copies per liter (gc/L) ranged from 2.1 × 10^2^ to 3.1 × 10^5^ gc/L and varied based on the sample volume and qPCR dilution factor. NTM densities were higher in the river samples, with a median of 7.3 × 10^4^ gc/L (*n* = 11). Gene copy densities increased after filtration, from less than the LOQ in the Ozone Effluent to a median of 1.1 × 10^4^ gc/L in the Filter Effluent (*n* = 7). In the Finished Water samples, the median NTM gene copy density was 1.4 × 10^3^ gc/L (*n* = 8). At Sites A and B, maximum NTM densities in the first draw samples reached 5.5 × 10^5^ gc/L and 4.2 × 10^7^ gc/L, respectively.

In the building plumbing samples, the highest NTM densities were observed in the first draw samples (median: 6.7 × 10^4^ gc/L, *n* = 34), followed by the 5-min flush samples (median: 2.4 × 10^4^ gc/L, *n* = 34) and full flush samples (median: 2.1 × 10^4^ gc/L, *n* = 33). NTM in the full flush samples, which captured distribution system water quality at the building locations, ranged from a median of 5.3 × 10^3^ gc/L at Site E to 1.0 × 10^5^ gc/L at Site B. Differences in NTM densities were observed over the sampling months. The months with the lowest median NTM densities in first draw samples were November (1.1 × 10^4^ gc/L, *n* = 5) and December (3.4 × 10^4^ gc/L, *n* = 4), while the highest median NTM densities in the first draw samples were found in October (3.2 × 10^6^ gc/L, *n* = 2), May (1.9 × 10^5^ gc/L, *n* = 3), and January (1.3 × 10^5^ gc/L, *n* = 5). In the full flush samples, the months with the highest median NTM densities were March (3.3 × 10^4^ gc/L, *n* = 5) and January (3.0 × 10^4^ gc/L, *n* = 5), and the month with the lowest median NTM density was November (2.9 × 10^3^ gc/L, *n* = 5).

### NTM species identified by metagenomic analysis

**Fig 3:**
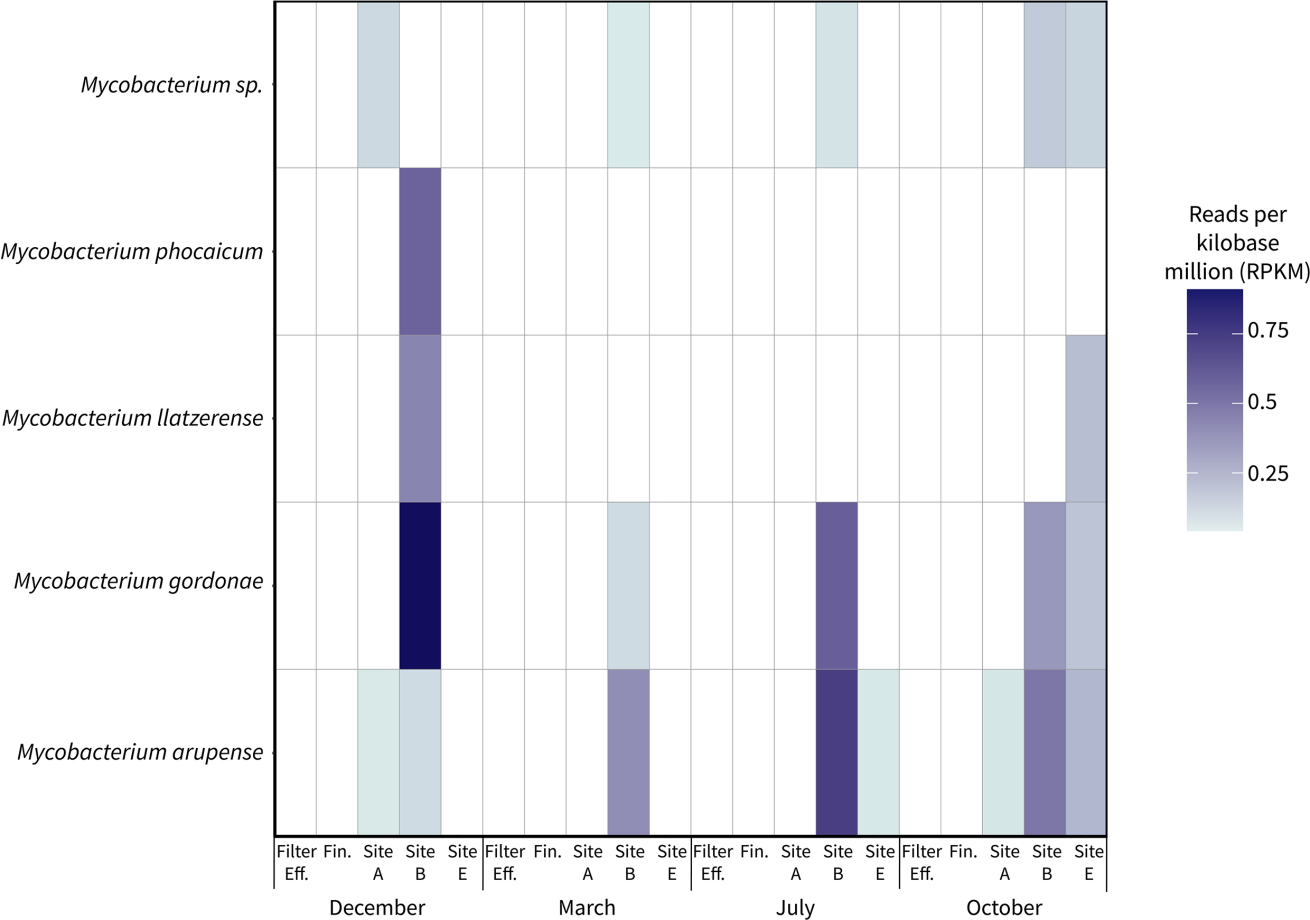
Metagenome-assembled genomes (MAGs) identified as *Mycobacterium*. Relative abundances of reads mapping to the NTM MAGs are represented by reads per kilobase million (RPKM). Filter Eff.: Filter Effluent; Fin.: Finished Water, Site A: Site A full flush sample, Site B: Site B full flush sample, Site E: Site E full flush sample.

Processing of the metagenomic sequences from the WTP filter effluent, finished water, and full flush samples yielded five high-quality (>50% completeness and <10% redundancy) NTM metagenome-assembled genomes (MAGs) (Fig. 3; Table S6). Of these, four were identified to the species level, representing *M. phocaicum*, *M. llatzerense*, *M. gordonae,* and *M. arupense*. The MAG with the highest relative abundance across all samples was the one identified as *M. gordonae*, which occurred at a relative abundance of 0.91 reads per kilobase million (RPKM) in the December Site B full flush sample. The *M. gordonae* MAG was also detected in the Site B full flush samples from the other months, with relative abundances ranging from 0.08 to 0.51 RPKM, and in the October Site E full flush sample (0.15 RPKM). The NTM MAG not identified to the species level was detected in the full flush samples from Site A in December, Site B in March, July, and October, and Site E in October, with relative abundances ranging from 0.04 to 0.14 RPKM. The only detection of the *M. phocaicum* MAG was in the December Site B full flush sample (0.49 RPKM). The highest relative abundance of the *M. arupense* MAG was in the July Site B full flush sample (0.65 RPKM). The *M. arupense* MAG was detected in the other Site B samples, as well as the Site A samples from December and October and the Site E samples from July and October. The *M. llatzerense* MAG was detected in the December Site B full flush sample (0.36 RPKM) and October Site E full flush sample (0.17 RPKM). No reads corresponding to the filter effluent or finished water samples met the 25% coverage minimum.

The highest relative abundances for all the MAGs except for the NTM MAG not identified to the species level occurred in the Site B full flush samples. However, the months with the highest observed relative abundances varied, with the highest relative abundances for the *M. phocaicum*, *M. llatzerense*, and *M. gordonae* MAGs occurring in December, while the highest relative abundance of the *M. arupense* MAG occurred in July. The December Site B and October Site E full flush samples were the only two samples in which four of the five MAGs were detected. While most of the MAGs were detected across seasons, *M. phocaicum* was only detected in December and *M. llatzerense* was only detected in December and October.

### Physicochemical and HPC analyses

**Fig 4:**
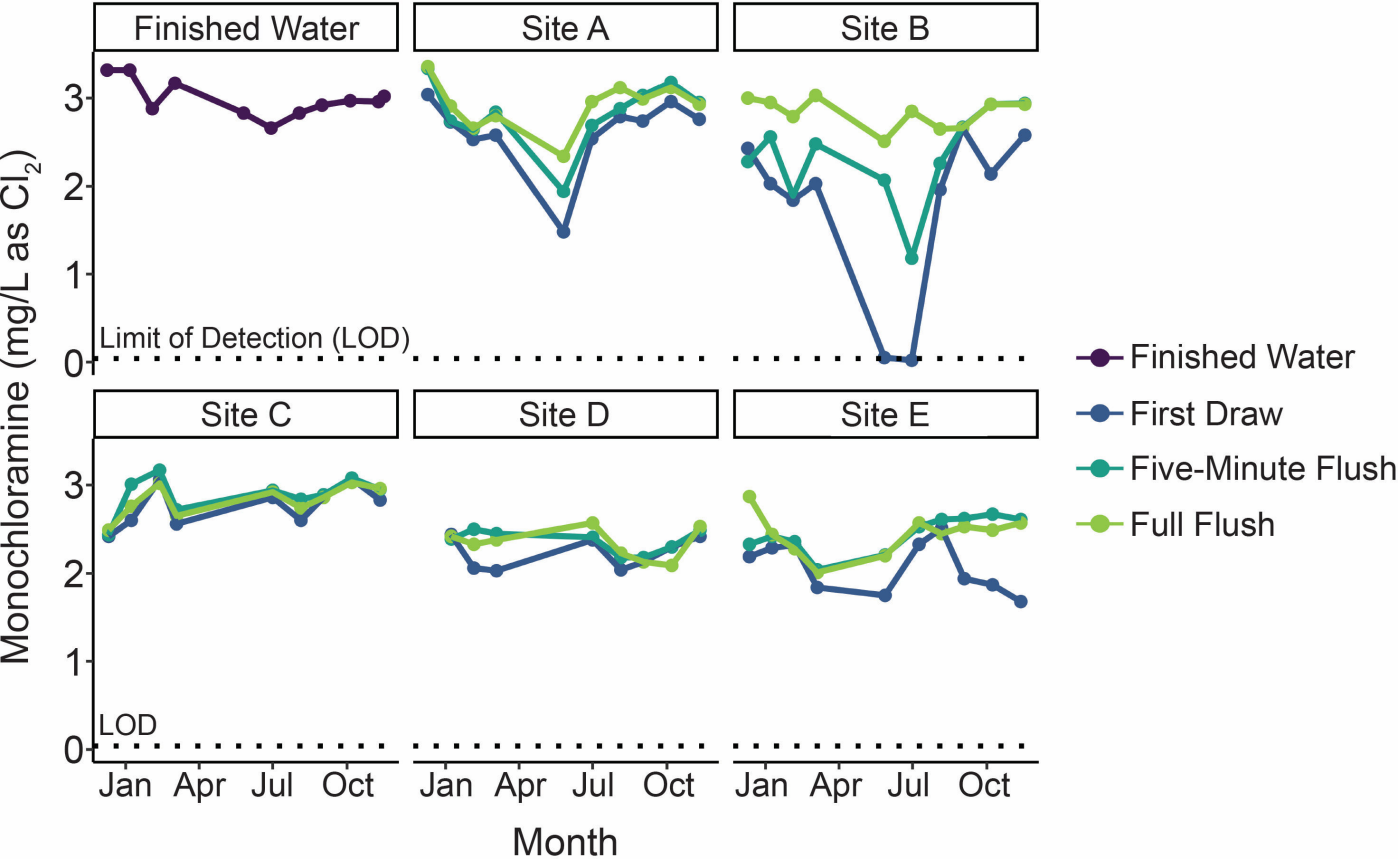
Monochloramine concentrations in the finished water and the five distribution system sites (Sites A–E). Samples at the distribution system sites included first draw, 5-min flush, and full flush. The dotted line indicates the limit of detection (LOD, 0.04 mg/L as Cl_2_). Based on data from Dowdell et al. (44).

Water sample temperatures fluctuated with the season, with temperatures ranging from less than 5°C in February 2020 to 24°C in August 2020 (Fig. S3). Although the Well-Source temperatures were relatively stable over the year (median: 11.9 ± 0.6°C, *n* = 9), Well-Plant temperature trends were influenced by season (median: 18.0 ± 5.0°C, *n* = 9). pH values did not vary substantially at any of the sampling locations over the sampling campaign (standard deviations ranged from <0.1–0.2; Fig. S4). Median pH values were 7.3 in the well samples (Well-Source and Well-Plant; *n* = 18), 8.1 in the river samples (River-Source and River-Plant; *n* = 14), and 9.3 across all other sampling locations (*n* = 166). Turbidities in the pre-filtration samples were typically greater than one nephelometric turbidity unit (NTU), with the highest turbidities occurring in the well water samples (median: 18.0 NTU, *n* = 18) and the Ozone Effluent (median:7.0 NTU, *n* = 9; Fig. S5). Median turbidity values for samples post-filtration were less than 0.3 NTU at all locations, except the first draw samples from Site E, where the median turbidity was 0.5 NTU (*n* = 10). Monochloramine concentrations in the finished water were approximately 3.0 mg/L across all sampling events (median: 3.0 mg/L as Cl_2_, *n* = 11; Fig. 4). Building water sample monochloramine concentrations varied by location, sample type, and month. Of the first draw building water samples (median: 2.4 mg/L as Cl_2_, *n* = 47), the lowest monochloramine concentrations were observed in the May and June samples at Site B, which were near or below the LOD of 0.04 mg/L as Cl_2_.

Nitrogen species (total ammonia, nitrate, and nitrite) were generally low, particularly in the source waters, treatment plant, and finished water samples. For total ammonia (LOD: 0.01 mg/L NH_3_-N), increased concentrations were observed in the Site B first draw and 5-min flush samples collected during the summer, with the highest concentration, 0.50 mg/L NH_3_-N, occurring in the July first draw sample (Fig. S6). Nitrate (LOD: 0.02 mg/L NO_3_-N) concentrations were less than 1.0 mg/L NO_3_-N in all samples except in the July Site A first draw and 5-min flush samples, where results were 2.3 mg/L NO_3_-N and 1.8 mg/L NO_3_-N, respectively (Fig. S7). Nitrite (LOD: 0.002 mg/L NO_2_-N) was also low across most sites, with most results less than 0.05 mg/L NO_2_-N (Fig. S8). However, higher nitrite concentrations were observed in the March Filter Effluent (0.08 mg/L NO_2_-N), the July Site B first draw (0.10 mg/L NO_2_-N), all samples from Site D collected in August through October (maximum: 0.16 mg/L NO_2_-N in the August first draw sample), and the November Site E first draw sample (0.11 mg/L NO_2_-N).

HPC results were generally low after secondary disinfection, except in some first draw samples (Fig. S9). River water HPC results ranged from 1.5 × 10^3^ to 6.3 × 10^3^ CFU/mL (median: 3.9 × 10^3^ CFU/mL, *n* = 7). HPC results were lower in the well water samples, which ranged from <1 CFU/mL to 8.3 × 10^2^ CFU/mL (median: 6.4 × 10^2^ CFU/mL, *n* = 16). Notably, the HPC results were significantly higher in the Well–Plant samples compared to the Well–Source samples (*P* < 0.01), indicating bacterial growth during transmission of the well water to the WTP. Ozone Effluent HPC results ranged from 5 to 1.0 × 10^3^ CFU/mL (median: 1.2 × 10^2^ CFU/mL, *n* = 8). Filter effluent HPC results were generally higher than in the ozone effluent, ranging from 18 to 2.6 × 10^4^ CFU/mL (median: 1.0 × 10^4^ CFU/mL, *n* = 8). HPC results in the finished water ranged from 1 to 30 CFU/mL (median: 4 CFU/mL, *n* = 8). In the distribution system, the full flush samples had the lowest HPC results overall (median: 8 CFU/mL, *n* = 45) while the first draw samples were the highest (median: 1.7 × 10^2^ CFU/mL, *n* = 45). However, median HPC results for first draw samples varied by site, with median results at Sites A (4.0 × 10^3^ CFU/mL, *n* = 9), B (4.4 × 10^3^ CFU/mL, *n* = 10), and E (median: 1.8 × 10^2^ CFU/mL, *n* = 10) being at least one order of magnitude higher than the median HPC results at Sites C (41 CFU/mL, *n* = 8) and D (4 CFU/mL, *n* = 8).

### Impacts of decreased water use and flushing on physiochemical parameters and NTM

**Fig 5:**
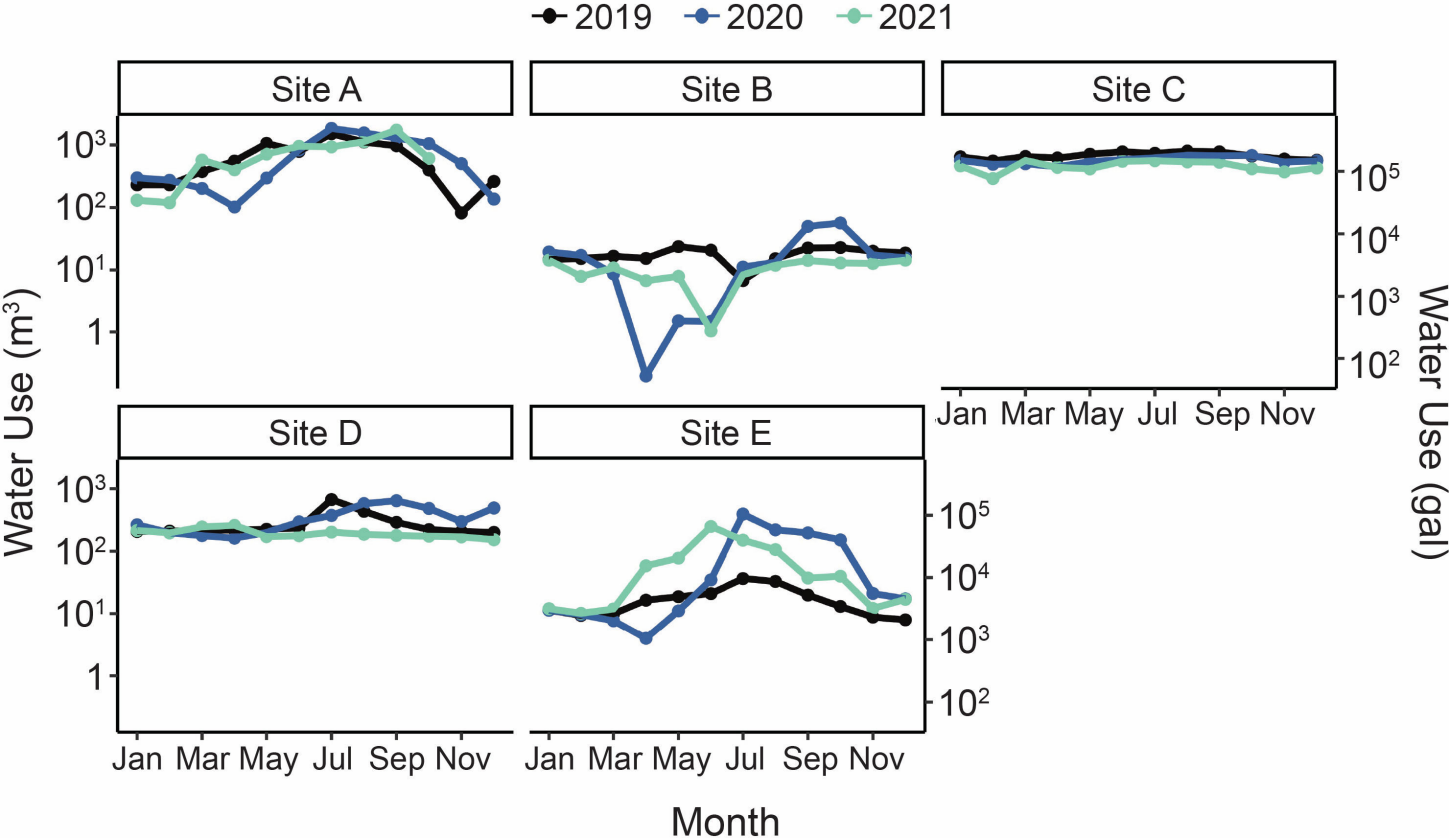
Monthly building water usage in the years 2019, 2020, and 2021. Units are cubic meters (left *y*-axis) and gallons (right *y*-axis).

The COVID-19 pandemic occurred during sampling, resulting in lower than usual water usage in several distribution system monitoring locations beginning in mid-March 2020 (Fig. 5). At Site A, the monthly water use in March, April, and May 2020 was at least 46% lower than in 2019, returning to values similar to 2019 beginning in June 2020. At Site B, March 2020 water use decreased to 48% of 2019 use, with the lowest water use occurring from April through June 2020, which was at least 90% less than the monthly water use during those months in 2019. At Site B, water use rebounded beginning in July 2020. At Site E, monthly water use in 2020 was below 2019 levels from March through May 2020 but then was substantially higher in the remaining months of 2020.

The low water use at Sites A, B, and E was associated with decreased disinfectant residual (Fig. 4). Specifically, the monochloramine residuals in the first draw samples from Sites A and B in May 2020 were at least 50% lower than the monochloramine residuals in the March samples, which were collected prior to building closures. At Site B, first draw monochloramine residuals were near or below the detection limit (0.04 mg/L as Cl_2_) in both May and July. At Site B, first draw samples during the low water use period also had higher turbidities (maximum: 4.6 NTU in the July first draw sample) than the first draw samples taken once water use increased to normal levels (Fig. S5).

Tap flushing improved water quality parameters at several building plumbing locations though the degree of improvement and amount of flush time required for improved conditions varied by building and parameter. Flushing significantly reduced NTM qPCR results at Sites A and B (first draw vs full flush, *P* < 0.05) but did not significantly reduce NTM qPCR results at Site C, D, and E. The flush duration required to reduce NTM qPCR results at Sites A and B varied. At Site A, the NTM densities in the 5-min flush and full flush were similar (*P* > 0.05), suggesting that distribution system water was reached after 5 min flushing, or that building plumbing NTM levels were similar to distribution system levels. In contrast, flushing beyond 5 min reduced NTM gene copies by an average of 0.7 log at Site B.

### Influence of water age and water quality parameters on NTM

To investigate the influence of distribution on NTM and physicochemical parameters, correlation analysis was performed. The correlation analysis included temperature, pH, turbidity, monochloramine concentration, estimated water age at the site, and the NTM qPCR results for the full flush samples (*n* = 32). As this correlation analysis was focused on the distribution system water age, first draw and 5-min samples were not included. The results showed that estimated water age had a strong negative correlation with monochloramine concentration (Kendall’s tau: −0.50, *P* < 0.01; Fig. S10). Estimated water age was also positively correlated with turbidity though the association was weaker (Kendall’s tau: 0.24, *P* > 0.05). Total NTM were not strongly associated with any of the water quality parameters though a weak positive correlation was observed with pH (Kendall’s tau: 0.23, *P* > 0.05). The relationship between HPC and NTM qPCR results was also investigated. For all samples, HPC results were significantly but weakly correlated with NTM (*n* = 141, Kendall’s tau: 0.18, *P* < 0.01). When only considering distribution system samples, the correlation was slightly stronger (*n* = 103, Kendall’s tau: 0.28, *P* < 0.01).

## DISCUSSION

This study is unique in that monitoring had started several months prior to the COVID-19 pandemic, which allowed for the evaluation of how decreased water use in buildings impacted general water quality parameters and NTM. At Sites A and B, where buildings were closed in mid-March 2020, lower water qualities were observed in first draw samples, including turbidities as high as 4.6 NTU, HPC results as high as 8.0 × 10^4^ CFU/mL, and one sample without a measurable monochloramine residual. In addition to general water quality impacts, the NTM gene copy densities were also higher, with values as high as 4.2 × 10^7^ gc/L. These NTM densities were similar to those reported in another study that investigated the impact of pandemic-related building closures on NTM using qPCR (17). As the buildings re-opened and water use increased in late summer and fall 2020, the NTM densities and physicochemical parameters, including monochloramine residual, generally improved. At Sites C and D, which remained fully open during the pandemic lockdown, water use was maintained as usual. This continuation of similar water use patterns was reflected in the consistency of the NTM densities and monochloramine residuals across the year of sampling. Although most studies investigating the impacts of COVID-19 pandemic-related building closures focused on other parameters, such as metals or *L. pneumophila* (33–35, 82), this study showed the importance of also considering the impact of low water use on NTM occurrence.

By sampling from drinking water sources to taps in buildings in the distribution system, this study investigated how NTM populations change through water treatment and delivery. In well water, NTM gene copy densities changed even prior to treatment, increasing during conveyance from the well to the WTP. Transmission of well water to the WTP also significantly increased water temperatures (*P* < 0.05; Fig. S3) and HPC results (*P* < 0.05, Fig. S9) in the Well-Plant samples compared to the Well-Source samples. As a result, the temperature curve for the Well-Plant samples is more similar to that of the surface water samples than the Well-Source samples (Fig. S3). While ozonation effectively reduced NTM gene copy densities to below the LOQ, these densities rebounded after filtration (median: 1 × 10^4^ gc/L) and only decreased after secondary disinfection in certain months. These findings support previous studies, which observed that NTM densities increased after filtration (26, 83) and that filtration shapes bacterial communities in the finished water (84).

Plate culture analyses indicated that the WTP samples were dominated by non-NTM, including spore-forming bacteria and microorganisms that could not be identified using MALDI-TOF MS. The first draw samples from Sites A and B and the full flush sample from Site B also yielded either all or a majority of non-NTM isolates. The WTP Filter Effluent was the first location where NTM isolates were recovered from culture plates and, except for the Site A first draw samples and Site B samples, the building plumbing sample isolates were at least 75% NTM. The low recovery of NTM isolates prior to filtration is likely due to high densities of non-NTM microorganisms in those samples. In previous studies focused on NTM quantification in source waters or varied water types, antibiotics were generally added to the NTM culture media in addition to using decontamination strategies (24, 39). Therefore, only using decontamination, as was done in this study, was insufficient for the recovery of NTM in certain samples with high densities of non-target microorganisms. However, the selected culture method and similar methods utilizing cetylpyridinium chloride (CPC), a common biocide, as a pre-treatment have been commonly used to quantify NTM in drinking water samples, including building water samples (23, 31, 36), and the method selected in this study performed well at all sites post-disinfection except for the first draw samples from Site A and the Site B samples.

The occurrence of specific species of NTM varied by site, with *M. aurum* only isolated from the WTP Filter Effluent, *M. llatzerense* only recovered from Site A, *M. avium* only recovered from Sites D and E, and *M. arupense, M. asiaticum*, and *M. gordonae* only isolated from Site E. The finding that *M. avium* was only recovered from the sites with the highest water ages supports the results of a previous study, which reported that the relative abundance of *M. avium* increased with water age (15). Other NTM species, including *M. chelonae* complex, *M. mucogenicum*/*phocaicum* group, and *M. franklinii,* were isolated from across sites with varying water ages. Overall, the NTM isolates (*n* = 194) were predominantly *M. chelonae* complex (43%, *n* = 84) and *M. mucogenicum/phocaicum* group (34%, *n* = 65). A recent study investigating NTM species in chloraminated distribution systems also reported that *M. mucogenicum/phocaicum* group was the most common NTM species recovered though in that study the proportion was higher at 76% of the NTM isolates (22).

Among the NTM isolates recovered in this study, *M. avium* and *M. chelonae* complex are most associated with human infection (85). While *M. avium* was only isolated from the full flush samples from Site D (*n* = 5) and Site E (*n* = 3), *M. chelonae* complex was the most commonly recovered isolate, representing 26% of all isolates and 43% of NTM isolates. *M. mucogenicum*, *M. phocaicum*, and *M. peregrinum* in drinking water have also been reported to cause human infections (58, 86–95). The prevalence of clinically relevant NTM species other than *M. avium* emphasizes the importance of monitoring for additional NTM species, such as those identified in this study, when investigating NTM occurrence in drinking water. Furthermore, studies have reported geographical differences in the NTM that account for the majority of infections (50, 57), which should be considered when selecting species-specific methods for NTM quantification.

Metagenomic analysis detected four species of NTM in the samples analyzed: *M. phocaicum*, *M. gordonae*, *M. llatzerense*, and *M. arupense*, all of which were also recovered by plate culture. However, there were differences in where these NTM species were detected when using plate culture versus metagenomic analysis. *M. phocaicum*, which was detected in the December Site B full flush sample by metagenomic analysis, was isolated by plate culture from all distribution system sampling sites (as the *M. mucogenicum/phocaicum* group). *M. arupense*, which was detected at Sites A, B, and E by metagenomic analysis, was only recovered from Site E with plate culture. Similarly, *M. gordonae* was detected in Site B and E samples by metagenomic analysis but only in Site E by plate culture, and *M. llatzerense* was detected in Sites B and E using metagenomic analysis but only in Site A by plate culture. Therefore, both methods detected certain species that were not identified using the other method at particular sites. Possible reasons for this disagreement include that the sequencing depth may have been insufficient to detect rarer species, that not all microbial community diversity was captured during binning, that the smaller sample volumes associated with the plate culture method may have prevented the detection of rarer species, and that the plate culture method may have de-selected for certain species. Also, the sequencing results include DNA from viable and non-viable microorganisms, whereas the plate culture results represent only viable and culturable microorganisms. These results highlight the benefits of using both culture-dependent and -independent methods, as it allows for a more complete understanding of NTM occurrence.

Although the culture-based method resulted in the identification of a larger number of NTM species, there are known biases associated with NTM plate culture methods. The pre-treatment step, for example, has been shown to impact species and strains of NTM differently, with one study reporting that a dose of 0.1% CPC for 30 min reduced culturable water-grown *M. avium A5* by approximately 80% but only reduced *M. avium Va14 (T*) by 20% (37). Differences in susceptibility of NTM species to pre-treatments by growth category (rapid-growing or slow-growing) have also been observed, with one study reporting that rapidly growing NTM species such as *M. fortuitum*, *M. abscessus*, and *M. peregrinum* may be more sensitive to decontamination (38). However, it is difficult to balance the need to reduce colony formation by non-NTM with recovery of target species. A new NTM plate culture medium that can be used without sample pre-treatment has been described recently (96). This method may overcome some of the limitations of existing methods, though additional comparisons are needed to validate its selectivity (96, 97). Similar to pre-treatment and media selection, incubation temperature may also influence NTM recovery. A review of studies culturing environmental NTM found that, while incubation temperatures vary widely, the average incubation temperature used was 35.6°C (98). In this study, we selected an incubation temperature of 35°C based on the available literature. Though Thomson et al. (2008) reported that differences were not observed when culturing *M. avium* and *M. intracellulare* at 32°C vs 35°C (36), differences in NTM recovery may occur when studies employ a wider range of temperatures.

Sampling over the course of a year allowed for investigation of the impacts of temperature and season on NTM populations. While strong seasonal trends were not observed, the NTM gene copy densities in full flush samples across all sites were lowest in November (median: 2.9 × 10^3^ gc/L, *n* = 5) and highest in March (3.3 × 10^4^ gc/L, *n* = 5). The impact of season could not be addressed for the first flush and 5-min flush samples due to the changes in water use patterns at several of the sites due to the COVID-19 pandemic. The impact of season on NTM species occurrence also could not be determined based on the MADLI-TOF MS results due to the low numbers of isolates analyzed per site per month and the strong influence of site on NTM species. The metagenomic analysis found the highest relative abundances of NTM MAGs in December and July. Overall, low water use appeared to be the driving factor of NTM densities in building plumbing rather than season. While few studies have investigated the impact of season on NTM, one previous study reported higher NTM gene copy densities in a distribution system during the summer compared to the winter (99). However, another study reported significantly higher NTM densities in distribution systems in winter and spring, which it attributed to lower water use (20). Due to COVID-19 pandemic-associated building closures during this study, it is difficult to determine whether the generally higher NTM densities observed in the summer are linked to season or were the result of shifts in water use patterns. Additional studies are needed to further investigate the potential impacts of both water use and season on NTM in drinking water distribution systems.

## MATERIALS AND METHODS

### Source water and treatment plant

The full-scale WTP sampled in this study has been described previously (16, 28, 84). Briefly, the City of Ann Arbor WTP is a 50 million gallon per day (1.9 × 10^5^ m^3^/day) facility in Ann Arbor, Michigan, USA, that treats groundwater and river water, which are blended at a ratio of approximately 1:6, using two-stage excess lime softening, coagulation, flocculation, sedimentation, ozonation, biological filtration, and monochloramine disinfection (3 mg/L as Cl_2_). Ozonation is used to achieve 0.5-log removal of *Cryptosporidium*. Typical ozone concentration × time (CT) values range from 0.3 to 1 mg/L. The biological filters contain either dual media sand- GAC or GAC alone. In June 2020, the WTP began using an ultraviolet (UV) disinfection system intermittently, which was intended to provide additional *Cryptosporidium* inactivation when the plant is operated with single-stage lime softening during maintenance periods. The UV system was in operation during the June, August, and September sampling events.

### Sample collection

Samples were collected approximately monthly from December 2019 through November 2020. Samples were not collected due to the COVID-19 pandemic in April 2020, and only a partial sampling event (finished water reservoir [Finished Water] and three buildings in the distribution system [Sites A, B, and E]) occurred in May 2020 due to building access restrictions. Samples were collected using sterile polypropylene bottles. All source water and treatment plant samples except the Ozone Effluent samples were either collected from the sampling taps that were continuously flowing (River-Plant, Well-Plant, and Finished Water) or were collected after flushing the taps for at least 5 min (River-Source, Well-Source, and Filter Effluent). Source water samples were collected from sampling taps at the source water pump stations (River-Source and Well-Source) as well as from the sampling taps at the WTP used for compliance monitoring (River-Plant and Well-Plant). River sample volumes ranged from 2 to 4 L and well samples ranged from 10 to 15 L. The Ozone Effluent samples were collected from a clearwell after the ozone contactors and prior to filtration. Ozone Effluent sample volumes ranged from 10 to 20 L. Filter Effluent samples were the combined effluents of six filters and ranged in volume from 10 to 20 L. Finished Water samples were collected from a sampling tap in the laboratory of the WTP used for compliance monitoring.

Cold water samples were collected from sinks in five buildings, Sites A – E, receiving water from the WTP via the distribution system (Table S1). These locations are a subset of the 13 used for distribution system compliance monitoring. Sites were selected to capture the full range of estimated water ages at compliance sites, as determined using an EPANET distribution system model (100). Sites were ordered based on estimated water age and selected to capture a range of water quality characteristics based on historical data provided by the WTP, which included concentrations of total chlorine and nitrogen species, and HPC. Additional details on the distribution sampling locations are included in Table S1. Three samples were collected from each distribution sampling location: (i) a 2 L first draw sample, representing the water in the fixture and building plumbing line supplying the fixture, (ii) a 10 L 5-min flush sample, meant to represent the typical monitoring sample collected by the utility, and (iii) a 10–20 L fully flushed sample, meant to represent distribution system water at the location. To mimic the procedure for compliance sampling, the tap was not disinfected for the first draw samples but was sprayed with a 10% bleach solution prior to collection of the 5-min flush and full flush samples. For the full flush samples, temperature and pH were monitored and samples were collected when successive readings stabilized.

### Physicochemical analyses

Temperature, pH, conductivity, and total dissolved solids (TDS) were analyzed immediately on-site using a combination probe (Hanna Instruments HI98121, Smithfield, RI, USA). Total chlorine, free chlorine, monochloramine, total ammonia (i.e., the sum of ammonium and ammonia), and nitrite concentrations were also analyzed on-site using a portable colorimeter (Hach DR900, Loveland, CO, USA) and powder pillows (Hach Methods 10250, 10245, 10200, 10200, and 8507). Samples for total organic carbon (TOC) were transferred to carbon-free glass vials and acidified with 85% phosphoric acid. TOC samples were analyzed with the non-purgeable organic carbon (NPOC) method using a Shimadzu TOC analyzer (TOC-V, Japan). Turbidity was measured using a benchtop turbidimeter (Hach TU5200). Nitrate concentrations were quantified using a Dionex ICS-2100 ion chromatography system following US EPA Method 300.1 (101).

### DNA collection and extraction

Water samples were filtered onto sterile 0.22-µm copolymer cartridge filters (EDM Millipore, USA, cat. no. SVGPL10RC) using sterile tubing (Masterflex, USA) and peristaltic pumps. Filters were then placed in sterile bags. Samples collected from December 2019 through March 2020 were filtered in the field and flash frozen using dry ice and ethanol and then transported to the laboratory and frozen at −80°C. Due to COVID-19 pandemic restrictions, samples collected after March 2020 were transported in coolers with cold packs to the laboratory for filtration and were placed in the −80°C freezer rather than flash freezing with dry ice.

DNA was extracted using a modified form of the QIAGEN DNeasy PowerWater kit (QIAGEN, Hilden, DEU, cat. no. 14900-100-NF) method, which includes additional enzymatic (Proteinase K and lysozyme) and chemical (chloroform-isoamyl 24:1) lysis steps (102). DNA purity was measured using a Nanodrop 1.0 spectrophotometer (Thermo Fisher Scientific, Waltham, MA, USA), and DNA concentrations were measured using the Qubit dsDNA High-Sensitivity assay kit (Thermo Fisher Scientific, cat. no. Q32851).

### NTM and HPC culture methods

Samples for HPC analyses were transferred to 100 mL sterile vessels containing excess sodium thiosulfate and analyzed using the pour-plate method (SM 9215-B-2000) (103). NTM plate culture was performed for all sampling events except February, May, and August 2020. The residual disinfectant was not quenched in NTM plate culture samples due to the potential for sodium thiosulfate to impact NTM recovery (36). Samples were processed on the same day as sampling, often within a few hours of sample collection, to limit the impact of residual disinfectant. Sample volumes for NTM plate culture ranged from 3 mL to 1 L and were adjusted each month based on colony counts for the previous month in an effort to maximize the number of plates that yielded 1–300 colonies (Table S3). Samples (*n* = 148) were processed in duplicate except when insufficient volume was available to process in duplicate (occurred in 13 cases, all of which were first draw samples with limited volume). Filter concentration followed by pre-treatment with CPC was employed. Briefly, samples were transferred to sterile glass bottles and dosed with a sterile 2% CPC solution to a final concentration of 0.04%. Samples were incubated at room temperature for 30 min and then immediately filtered onto 0.45-µm filters (Thermo Fisher Scientific, cat. no. 09–719-555) using sterile glass membrane filtration funnels and bases and a vacuum filtration manifold (EDM Millipore, USA) in a fume hood. After filtration, the filters were rinsed with 50 mL of sterile ultrapure water (Invitrogen UltraPure DNase/RNase-Free Distilled Water, Thermo Fisher Scientific, cat. no. 10977015) to remove residual CPC. Filters were plated onto sterile Middlebrook 7H11 agar (Thermo Fisher Scientific, cat. no. R454002) supplemented with oleic albumin dextrose catalase (OADC, BBL Middlebrook OADC Enrichment, BD Biosciences, Franklin Lakes, NJ, USA, cat. no. 212351) using sterile tweezers in a biological safety cabinet. Plates were allowed to dry in the biological safety cabinet, sealed with Parafilm (Bemis Company, USA), inverted, and placed in loosely sealed plastic bags. Plates were incubated in the dark at 35°C for at least 1 month and observed at least once per week. Controls were included for at least every sampling day and included plate controls (agar plates only, *n* = 24) and filtration controls (50 mL of ultrapure water filtered and plated, *n* = 26).

Plates were classified into four groups, either negative (no colonies), positive and quantifiable (CFU between 1 and 300), too numerous to count (TNTC; CFU greater than 300), or no count (NC). The NC category represented plates that contained spreader colonies or other colonies that grew to overtake the plates, preventing accurate counting of colonies, which may have originated in the samples or may have been the result of contamination. For calculations and plotting, TNTC plates were set to 300 CFU and negative plates were set to 0.5 CFU. Colony counts from the duplicate plates were averaged to obtain a plate count for each sample except when one of the plates was NC, in which case the NC plate was excluded. Excluding controls, a total of 284 plates were processed for 148 samples. Of these, 33 plates (12%) were TNTC, 44 (15%) were negative, and 20 (7%) fell in the NC category, leaving 187 plates (66%) from 107 (72%) samples that were counted. Only four of the 50 controls showed contamination. NC plates were not included in downstream analyses. For all plates that were counted, at least 10% of the colonies were picked for confirmation testing. Colonies were selected to cover a range of morphologies and growth characteristics. Colonies were picked in a biological safety cabinet using sterile pipette tips. Biomass was then transferred to sterile 0.6 mL tubes containing 50 µL of ultrapure water and frozen at −80°C.

### NTM isolate protein extraction MALDI-TOF MS analysis

Colonies (*n* = 392) from the NTM culture plates from the December, January, March, June, and October sampling events were analyzed using MALDI-TOF MS. Due to the number of isolates, only isolates from first draw and full flush samples were selected for analysis. Briefly, colonies were thawed and used to inoculate 5 mL tubes containing Middlebrook 7H9 with Tween 80 (Hardy Diagnostics, Santa Maria, CA, USA, cat. no. C62) and incubated at 35°C. Once growth was observed, isolates were streaked onto Middlebrook 7H11 with OADC and incubated at 35°C. Isolates that failed to grow on Middlebrook 7H11 with OADC at 35°C were streaked onto a Middlebrook 7H11 with OADC and incubated at 32°C and streaked onto an LB agar and incubated at 35°C. Seventy of the isolates (18%), from 25 samples, failed to grow despite the use of multiple temperatures and media and, therefore, could not be analyzed using MALDI-TOF MS.

For the protein extraction, each isolate was extracted in duplicate by picking 1 μL loopfuls of colonies from the culture plates and transferring them to two 2 mL tubes containing 200 µL of 0.5 mm zirconium/silica beads and 500 µL of 70% ethanol. Beads were heat-treated prior to use by incubating in a 450°C oven for at least 3 h. Tubes were vortexed for 15 min, then centrifuged at 10,000 × *g* for 2 min. The supernatant was discarded, and tubes were allowed to dry in a biosafety cabinet. Once dry, tubes were transferred to a fume hood, where 20 µL of 70% formic acid was added to each tube. Tubes were vortexed and incubated for 5 min, and then 20 µL of acetonitrile was added. Tubes were centrifuged again at 10,000 × *g* for 2 min, and the supernatant was used for MALDI-TOF MS analysis within 4 h of extraction. Each protein extraction session included a negative control (an empty bead tube), a non-NTM positive control (colonies of *Escherichia coli*), and an NTM positive control (colonies of *Mycobacterium smegmatis*).

MALDI-TOF MS analysis was performed at the University of Michigan Microbiome Core using a Bruker MALDI Biotyper sirius system (Bruker Daltonics, Billerica, MA, USA) and a 96 target brushed steel plate. Each MALDI-TOF MS plate analysis began with analyzing two targets of Bacterial Test Standard (BTS, Bruker Daltonics, Billerica, MA, USA, cat. No. 8255343) to ensure the system was functioning properly. All extracts were spotted in duplicate, and spectra were analyzed using both the standard instrument library and the *Mycobacterium* genus-specific Mycobacteria RUO Library (Bruker Daltonics). BTS scores were required to be above 2.0, and *M. smegmatis* positive control results were required to be above 1.7. The maximum score between the duplicate extractions and duplicate spots (four results per isolate) was used for identification. Minimum scores for identification followed manufacturer guidance; NTM isolates were required to score at least 1.6 to be identified to the species or complex level, while non-NTM isolates were required to score at least 1.7 (69). Per the manufacturer, NTM isolates with scores less than 1.6 were not identifiable, isolates with scores between 1.60 and 1.79 were “low confidence” identifications, and isolates with scores of at least 1.8 were considered “high confidence” identifications. Score results by genus and species are provided in Table S4. Due to the inability of the method to distinguish between *M. chelonae*, *Mycobacterium stephanolepidis*, and *Mycobacterium salmoniphilum*, these species are reported as *M. chelonae* complex. The method also could not distinguish between *M. mucogenicum* and *M. phocaicum*, so matches are reported as *M. mucogenicum/phocaicum* group. The MALDI-TOF MS results for isolates were used to adjust the presumptive NTM CFU/mL by multiplying the presumptive CFU/mL by the percentage of the isolates from the sample that were identified as NTM.

### NTM qPCR

qPCR analysis followed the guidance of the Minimum Information for Publication of Quantitative Real-Time PCR Experiments (MIQE) and Environmental Microbiology Minimum Information Guidelines (EMMI) (104, 105). Gene copies of the *Mycobacterium atpE* gene were quantified using the Radomski et al. assay (FatpE: 5′-CGGYGCCGGTATCGGYGA-3′; RatpE: 5′-CGAAGACGAACARSGCCAT-3′) (42), generating an approximately 164 base pair amplicon, with the modification that EvaGreen was used instead of probe chemistry (16). qPCR was performed using 96-well plates containing 10 µL reactions composed of 5 µL of master mix (Biotium FastEvaGreen 2×, final concentration 1×, cat. no. 31003), 0.5 µL each 10 µM forward and reverse primers (final concentration of 0.5 µM, Integrated DNA Technologies [IDT], Coralville, IA, USA), 0.25 mL of 25 mg/mL bovine serum albumin (Thermo Fisher Scientific, cat. no. AM2616, final concentration 0.625 mg/mL), 2.75 µL of ultrapure water and 1 µL of template. Samples were analyzed using a real-time PCR system (Applied Biosciences QuantStudio 3, Thermo Fisher Scientific) using the following cycling conditions: 95°C for 5 min, 35 cycles of 95°C for 20 s, 59.6°C for 30 s, and 72°C for 30 s. Melt curve analysis was performed for all plates. Standard curves were prepared using synthetic DNA (gBlock, IDT, USA), consisting of the amplicon with 30 base pairs of neutral adaptors on both ends. Standard curves were included on each 96-well plate. Negative controls with ultrapure water in place of template were also included on each 96-well plate. The LOD (41 gc/rx) and LOQ (41 gc/rxn) were determined using serial dilutions of the standards. Samples were diluted to reduce inhibition, with dilution factors ranging from undiluted to 1:110. Inhibition was assessed on the majority of samples (88%, *n* = 141) by analyzing samples at two dilution levels and comparing the actual Cq to the theoretical Cq. Thresholds were automatically set by the qPCR instrument. All plates were required to meet a minimum efficiency of 85% and *R*^2^ standard curve results of at least 0.98. Results were converted from gc/rxn to gc/L using the volume of template per reaction (1 µL), the dilution factor, the DNA extraction elution volume (100 µL), and the filtered sample volume.

### Metagenomic analysis

The Filter Effluent, Finished Water, and full flush samples from distribution system Sites A, B, and E from the December, March, June, and October sampling events (*n* = 20) and three negative controls (pooled blank filter controls, filtration controls, and DNA extraction controls) were submitted for shotgun metagenomic sequencing. Library preparation, sequencing, and de-multiplexing were performed by the University of Michigan Advanced Genomics Core. Paired-end sequence libraries were prepared using the NEBNext Ultra II FS DNA Library Prep Kit for Illumina (New England BioLabs Inc., Ipswich, MA, USA, cat. no. E7805S). Sequencing was performed using the NovaSeq 6000 system and an SP flow cell with 500 cycles, producing 250 nucleotide paired-end reads.

Bioinformatic analysis of reads followed the pipeline described in Vosloo et al. (106). Briefly, pre-processing was performed using fastp (v0.20.0) (107) to remove adaptors and perform initial quality filtering. Reads mapping to the UniVec_Core database (ftp://ftp.ncbi.nlm.nih.gov/pub/UniVec/) and negative controls were removed as potential contamination. The cleaned reads were pooled and *de novo* co-assembly into contigs was performed using metaSPAdes (v.3.13.1) with kmer sizes of 21, 33, 55, 77, 99, and 127 (108). Contigs less than 1 kilobase pair were removed using seqtk (https://github.com/lh3/seqtk), and redundant contigs were removed using the *dedupe* function of BBTools (v38.76) (109). The quality of the assembly was determined using QUAST (v.5.0.2) (110), and assembly validation was performed by calculating the mapping rate of processed reads to contigs. Binning was performed using Anvi’o (v6.1) workflow for the analysis and visualization of omics data. Binning algorithms CONCOCT (v.1.1.1), MetaBAT (v.2.12.1), and MaxBin (v.2.2.4) (111–114) were used together with the bin aggregation software DAS Tools (v.1.1.0) to select the highest quality bins with the least redundancy (115). Bin statistics, including bin size, GC content, and number of contigs, were determined using the summarize function in Anvi’o. Further bin quality estimates, including completeness, redundancy, and strain heterogeneity, were determined using CheckM (v 1.0.18) (116). To improve bin quality, bins with at least 50% completeness were reassembled using metaSPAdes with the same kmer sizes previously used. Re-binning was performed using the same binning strategies and manually curated using Anvi’o. Duplicate bins were dereplicated using dRep (v2.6.2) and clustered into species-level representative genomes using a 95% average nucleotide identity (117). The species-level representative genomes were classified using the Genome Taxonomy Data set Toolkit (GTDTk, v0.3.2) (118), and final bin statistics and quality were determined as described previously. CoverM (v0.4.0) was used to calculate coverage of the MAGs across samples with RPKM as a metric for relative abundance (119). The covered_bases parameter of coverM was also used to calculate the number of bases covered by one or more reads at a coverage threshold of 25%, indicating that only bases with sequencing read coverage equal to or greater than 25% of the expected coverage depth were considered covered.

### Data analysis

Data cleaning and analyses were performed using R (version 4.1.1) and R Studio (1.4.1717) (120, 121). R packages used for analyses include *ggplot, dplyr, lubridate, viridis, readxl,* and *stats*. For the qPCR data analysis and plotting, values less than the LOQ were set at one-half the LOQ. For physicochemical parameters, values less than the LOD were set at one-half the LOD. Statistical analyses were performed using the *stats* package. Differences between sample locations, sample types, and by season were calculated using the Wilcoxon signed-rank test using a significance threshold of 0.05. Correlation analysis and correlation plot were performed using the functions “cor” and “cor.test” from the R package *stats* and the R package *corrplot* using Kendall’s tau b.

## Data Availability

The raw sequencing data are available through the National Center for Biotechnology Information (NCBI) under BioProject no. PRJNA1081894.

## SUPPLEMENTAL MATERIAL

Supplemental material aem.00609-24-s0001.pdf Figures S1 to S10; Tables S1 to S6.

ASM does not own the copyrights to Supplemental Material that may be linked to, or accessed through, an article. The authors have granted ASM a non-exclusive, world-wide license to publish the Supplemental Material files. Please contact the corresponding author directly for reuse.

## References

[1] Park SC, Kang MJ, Han CH, Lee SM, Kim CJ, Lee JM, Kang YA. 2019. Prevalence, incidence, and mortality of nontuberculous mycobacterial infection in Korea: a nationwide population-based study. BMC Pulm Med 19:140. doi:10.1186/s12890-019-0901-z 31370826 PMC6670190

[2] Shah NM, Davidson JA, Anderson LF, Lalor MK, Kim J, Thomas HL, Lipman M, Abubakar I. 2016. Pulmonary Mycobacterium avium-intracellulare is the main driver of the rise in non-tuberculous mycobacteria incidence in England, Wales and Northern Ireland, 2007-2012. BMC Infect Dis 16:195. doi:10.1186/s12879-016-1521-3 27154015 PMC4858927

[3] Prevots DR, Loddenkemper R, Sotgiu G, Migliori GB. 2017. Nontuberculous mycobacterial pulmonary disease: an increasing burden with substantial costs. Eur Respir J 49:1700374. doi:10.1183/13993003.00374-2017 28446563 PMC11037024

[4] Collier SA, Deng L, Adam EA, Benedict KM, Beshearse EM, Blackstock AJ, Bruce BB, Derado G, Edens C, Fullerton KE, Gargano JW, Geissler AL, Hall AJ, Havelaar AH, Hill VR, Hoekstra RM, Reddy SC, Scallan E, Stokes EK, Yoder JS, Beach MJ. 2021. Estimate of burden and direct healthcare cost of infectious waterborne disease in the United States. Emerg. Infect. Dis 27:140–149. doi:10.3201/eid2701.190676 33350905 PMC7774540

[5] Greco SL, Drudge C, Fernandes R, Kim J, Copes R. 2020. Estimates of healthcare utilisation and deaths from waterborne pathogen exposure in Ontario, Canada. Epidemiol Infect 148:e70. doi:10.1017/S0950268820000631 32167443 PMC7118719

[6] Gerdes ME, Miko S, Kunz JM, Hannapel EJ, Hlavsa MC, Hughes MJ, Stuckey MJ, Francois Watkins LK, Cope JR, Yoder JS, Hill VR, Collier SA. 2023. Estimating waterborne infectious disease burden by exposure route, United States, 2014. Emerg Infect Dis 29:1357–1366. doi:10.3201/eid2907.230231 37347505 PMC10310388

[7] Mehta M, Marras TK. 2011. Impaired health-related quality of life in pulmonary nontuberculous mycobacterial disease. Respiratory Medicine 105:1718–1725. doi:10.1016/j.rmed.2011.08.004 21868209

[8] Dowdell K, Haig S-J, Caverly LJ, Shen Y, LiPuma JJ, Raskin L. 2019. Nontuberculous mycobacteria in drinking water systems - the challenges of characterization and risk mitigation. Curr Opin Biotechnol 57:127–136. doi:10.1016/j.copbio.2019.03.010 31003169 PMC6924000

[9] Nishiuchi Y, Iwamoto T, Maruyama F. 2017. Infection sources of a common non-tuberculous mycobacterial pathogen, Mycobacterium avium complex. Front Med (Lausanne) 4:27. doi:10.3389/fmed.2017.00027 28326308 PMC5339636

[10] Lande L. 2019. Environmental niches for NTM and their impact on NTM disease, p 131–144. In Griffith DE (ed), Nontuberculous mycobacterial disease: a comprehensive approach to diagnosis and management. Humana Press.

[11] Hamilton KA, Weir MH, Haas CN. 2017. Dose response models and a quantitative microbial risk assessment framework for the Mycobacterium avium complex that account for recent developments in molecular biology, taxonomy, and epidemiology. Water Research 109:310–326. doi:10.1016/j.watres.2016.11.053 27915187

[12] Proctor C, Garner E, Hamilton KA, Ashbolt NJ, Caverly LJ, Falkinham JO, Haas CN, Prevost M, Prevots DR, Pruden A, Raskin L, Stout J, Haig S-J. 2022. Tenets of a holistic approach to drinking water-associated pathogen research, management, and communication. Water Res 211:117997. doi:10.1016/j.watres.2021.117997 34999316 PMC8821414

[13] Ley CJ, Proctor CR, Singh G, Ra K, Noh Y, Odimayomi T, Salehi M, Julien R, Mitchell J, Nejadhashemi AP, Whelton AJ, Aw TG. 2020. Drinking water microbiology in a water-efficient building: stagnation, seasonality, and physicochemical effects on opportunistic pathogen and total bacteria proliferation. Environ Sci Water Res Technol 6:2902–2913. doi:10.1039/D0EW00334D

[14] Logan-Jackson AR, Flood M, Rose JB. 2021. Enumeration and characterization of five pathogenic Legionella species from large research and educational buildings. Environ Sci: Water Res Technol 7:321–334. doi:10.1039/D0EW00893A

[15] Haig S-J, Kotlarz N, LiPuma JJ, Raskin L. 2018. A high-throughput approach for identification of nontuberculous mycobacteria in drinking water reveals relationship between water age and Mycobacterium avium. mBio 9:e02354-17. doi:10.1128/mBio.02354-17 29440575 PMC5821076

[16] Haig S-J, Kotlarz N, Kalikin LM, Chen T, Guikema S, LiPuma JJ, Raskin L. 2020. Emerging investigator series: bacterial opportunistic pathogen gene markers in municipal drinking water are associated with distribution system and household plumbing characteristics. Environ Sci: Water Res Technol 6:3032–3043. doi:10.1039/D0EW00723D

[17] Hozalski RM, LaPara TM, Zhao X, Kim T, Waak MB, Burch T, McCarty M. 2020. Flushing of stagnant premise water systems after the COVID-19 shutdown can reduce infection risk by Legionella and Mycobacterium spp. Environ Sci Technol 54:15914–15924. doi:10.1021/acs.est.0c06357 33232602

[18] Lu J, Buse H, Struewing I, Zhao A, Lytle D, Ashbolt N. 2017. Annual variations and effects of temperature on Legionella spp. and other potential opportunistic pathogens in a bathroom. Environ Sci Pollut Res Int 24:2326–2336. doi:10.1007/s11356-016-7921-5 27815848 PMC6155451

[19] Aw TG, Scott L, Jordan K, Ra K, Ley C, Whelton AJ. 2022. Prevalence of opportunistic pathogens in a school building plumbing during periods of low water use and a transition to normal use. Int J Hyg Environ Health 241:113945. doi:10.1016/j.ijheh.2022.113945 35182850

[20] Zhang C, Struewing I, Mistry JH, Wahman DG, Pressman J, Lu J. 2021. Legionella and other opportunistic pathogens in full-scale chloraminated municipal drinking water distribution systems. Water Research 205:117571. doi:10.1016/j.watres.2021.117571 34628111 PMC8629321

[21] Rahmatika I, Simazaki D, Kurisu F, Furumai H, Kasuga I. 2023. Occurrence and diversity of nontuberculous mycobacteria affected by water stagnation in building plumbing. Water Supply 23:5017–5028. doi:10.2166/ws.2023.318

[22] Pfaller S, King D, Mistry JH, Alexander M, Abulikemu G, Pressman JG, Wahman DG, Donohue MJ. 2021. Chloramine concentrations within distribution systems and their effect on heterotrophic bacteria, mycobacterial species, and disinfection byproducts. Water Res. 205:117689. doi:10.1016/j.watres.2021.117689 34607086 PMC8682803

[23] Pfaller S, King D, Mistry JH, Donohue M. 2022. Occurrence revisited: Mycobacterium avium and Mycobacterium intracellulare in potable water in the USA. Appl Microbiol Biotechnol 106:2715–2727. doi:10.1007/s00253-022-11849-7 35298694 PMC9173748

[24] King DN, Donohue MJ, Vesper SJ, Villegas EN, Ware MW, Vogel ME, Furlong EF, Kolpin DW, Glassmeyer ST, Pfaller S. 2016. Microbial pathogens in source and treated waters from drinking water treatment plants in the United States and implications for human health. Sci Total Environ 562:987–995. doi:10.1016/j.scitotenv.2016.03.214 27260619

[25] Li Q, Yu S, Li L, Liu G, Gu Z, Liu M, Liu Z, Ye Y, Xia Q, Ren L. 2017. Microbial communities shaped by treatment processes in a drinking water treatment plant and their contribution and threat to drinking water safety. Front Microbiol 8:2465. doi:10.3389/fmicb.2017.02465 29312177 PMC5733044

[26] Le Dantec C, Duguet JP, Montiel A, Dumoutier N, Dubrou S, Vincent V. 2002. Occurrence of mycobacteria in water treatment lines and in water distribution systems. Appl Environ Microbiol 68:5318–5325. doi:10.1128/AEM.68.11.5318-5325.2002 12406720 PMC129932

[27] Thomas V, Loret J-F, Jousset M, Greub G. 2008. Biodiversity of amoebae and amoebae-resisting bacteria in a drinking water treatment plant. Environ Microbiol 10:2728–2745. doi:10.1111/j.1462-2920.2008.01693.x 18637950

[28] Kotlarz N, Rockey N, Olson TM, Haig S-J, Sanford L, LiPuma JJ, Raskin L. 2018. Biofilms in full-scale drinking water ozone contactors contribute viable bacteria to ozonated Water. Environ Sci Technol 52:2618–2628. doi:10.1021/acs.est.7b04212 29299927

[29] Donohue MJ, Vesper S, Mistry J, Donohue JM. 2019. Impact of chlorine and chloramine on the detection and quantification of Legionella pneumophila and Mycobacterium species. Appl Environ Microbiol 85:e01942-19. doi:10.1128/AEM.01942-19 31604766 PMC6881805

[30] Pryor M, Springthorpe S, Riffard S, Brooks T, Huo Y, Davis G, Sattar SA. 2004. Investigation of opportunistic pathogens in municipal drinking water under different supply and treatment regimes. Water Sci Technol 50:83–90. doi:10.2166/wst.2004.0025 15318491

[31] Donohue MJ, Mistry JH, Donohue JM, O’Connell K, King D, Byran J, Covert T, Pfaller S. 2015. Increased frequency of nontuberculous mycobacteria detection at potable water taps within the United States. Environ Sci Technol. 49:6127–6133. doi:10.1021/acs.est.5b00496 25902261

[32] Proctor CR, Rhoads WJ, Keane T, Salehi M, Hamilton K, Pieper KJ, Cwiertny DM, Prévost M, Whelton AJ. 2020. Considerations for large building water quality after extended stagnation. AWWA Water Sci 2:e1186. doi:10.1002/aws2.1186 32838226 PMC7323006

[33] Dowdell KS, Greenwald HD, Joshi S, Grimard-Conea M, Pitell S, Song Y, Ley C, Kennedy LC, Vosloo S, Huo L, Haig S-J, Hamilton KA, Nelson KL, Pinto A, Prévost M, Proctor CR, Raskin LM, Whelton AJ, Garner E, Pieper KJ, Rhoads WJ. 2023 Legionella pneumophila occurrence in reduced-occupancy buildings in 11 cities during the COVID-19 pandemic. Environ Sci: Water Res Technol. doi:10.1039/D3EW00278K

[34] Richard R, Boyer TH. 2021. Pre- and post-flushing of three schools in Arizona due to COVID-19 shutdown. AWWA Water Sci 3:e1239. doi:10.1002/aws2.1239 34901766 PMC8646703

[35] Rhoads WJ, Hammes F. 2021. Growth of Legionella during COVID-19 lockdown stagnation. Environ Sci: Water Res Technol 7:10–15. doi:10.1039/D0EW00819B

[36] Thomson R, Carter R, Gilpin C, Coulter C, Hargreaves M. 2008. Comparison of methods for processing drinking water samples for the isolation of Mycobacterium avium and Mycobacterium intracellulare. Appl Environ Microbiol 74:3094–3098. doi:10.1128/AEM.02009-07 18359837 PMC2394917

[37] Williams MD, Falkinham JO. 2018. Effect of cetylpyridinium chloride (CPC) on colony formation of common nontuberculous mycobacteria. Pathogens 7:79. doi:10.3390/pathogens7040079 30301158 PMC6313301

[38] Fernandes HMZ, Conceição EC, Gomes KM, da Silva MG, Dias RCS, Duarte RS. 2020. Recovery of non-tuberculous mycobacteria from water is Influenced by phenotypic characteristics and decontamination methods. Curr Microbiol 77:621–631. doi:10.1007/s00284-019-01704-w 31111226

[39] Radomski N, Cambau E, Moulin L, Haenn S, Moilleron R, Lucas FS. 2010. Comparison of culture methods for isolation of nontuberculous mycobacteria from surface waters. Appl Environ Microbiol 76:3514–3520. doi:10.1128/AEM.02659-09 20363776 PMC2876437

[40] Wang H, Bédard E, Prévost M, Camper AK, Hill VR, Pruden A. 2017. Methodological approaches for monitoring opportunistic pathogens in premise plumbing: a review. Water Res. 117:68–86. doi:10.1016/j.watres.2017.03.046 28390237 PMC5693313

[41] Aw TG, Rose JB. 2012. Detection of pathogens in water: from phylochips to qPCR to pyrosequencing. Curr Opin Biotechnol 23:422–430. doi:10.1016/j.copbio.2011.11.016 22153035 PMC7126744

[42] Radomski N, Roguet A, Lucas FS, Veyrier FJ, Cambau E, Accrombessi H, Moilleron R, Behr MA, Moulin L. 2013. atpE gene as a new useful specific molecular target to quantify Mycobacterium in environmental samples. BMC Microbiol. 13:277. doi:10.1186/1471-2180-13-277 24299240 PMC4219376

[43] Chern EC, King D, Haugland R, Pfaller S. 2015. Evaluation of quantitative polymerase chain reaction assays targeting Mycobacterium avium, M. intracellulare, and M. avium subspecies paratuberculosis in drinking water biofilms. J Water Health 13:131–139. doi:10.2166/wh.2014.060 25719473

[44] Dowdell KS, Raskin L, Olson T, Haig S-J, Dai D, Edwards M, Pruden A. 2022. Methods for detecting and differentiating opportunistic premise plumbing pathogens (OPPPs) to determine efficacy of control and treatment technologies. Report 4721. Denver, CO Water Research Foundation

[45] Lee E-S, Lee M-H, Kim B-S. 2015. Evaluation of propidium monoazide-quantitative PCR to detect viable Mycobacterium fortuitum after chlorine, ozone, and ultraviolet disinfection. Int J Food Microbiol 210:143–148. doi:10.1016/j.ijfoodmicro.2015.06.019 26143168

[46] Ditommaso S, Giacomuzzi M, Memoli G, Cavallo R, Curtoni A, Avolio M, Silvestre C, Zotti CM. 2020. Reduction of turnaround time for non-tuberculous mycobacteria detection in heater-cooler units by propidium monoazide-real-time polymerase chain reaction. J Hosp Infect 104:365–373. doi:10.1016/j.jhin.2019.10.010 31628958

[47] Nocker A, Sossa KE, Camper AK. 2007. Molecular monitoring of disinfection efficacy using propidium monoazide in combination with quantitative PCR. J Microbiol Methods 70:252–260. doi:10.1016/j.mimet.2007.04.014 17544161

[48] Gebert MJ, Delgado-Baquerizo M, Oliverio AM, Webster TM, Nichols LM, Honda JR, Chan ED, Adjemian J, Dunn RR, Fierer N. 2018. Ecological analyses of mycobacteria in showerhead biofilms and their relevance to human health. mBio 9:1–15. doi:10.1128/mBio.01614-18 PMC6212831 30377276

[49] Walsh CM, Gebert MJ, Delgado-Baquerizo M, Maestre FT, Fierer N. 2019. A global survey of mycobacterial diversity in soil. Appl Environ Microbiol 85:e01180-19. doi:10.1128/AEM.01180-19 31253672 PMC6696970

[50] Honda JR, Hasan NA, Davidson RM, Williams MD, Epperson LE, Reynolds PR, Smith T, Iakhiaeva E, Bankowski MJ, Wallace RJ, Chan ED, Falkinham JO, Strong M. 2016. Environmental nontuberculous mycobacteria in the Hawaiian Islands. PLoS Negl Trop Dis 10:e0005068. doi:10.1371/journal.pntd.0005068 27780201 PMC5079566

[51] Pinto AJ, Raskin L. 2012. PCR biases distort bacterial and archaeal community structure in pyrosequencing datasets. PLoS ONE 7:e43093. doi:10.1371/journal.pone.0043093 22905208 PMC3419673

[52] Bonk F, Popp D, Harms H, Centler F. 2018. PCR-based quantification of taxa-specific abundances in microbial communities: quantifying and avoiding common pitfalls. J Microbiol Methods 153:139–147. doi:10.1016/j.mimet.2018.09.015 30267718

[53] Crossette E, Gumm J, Langenfeld K, Raskin L, Duhaime M, Wigginton K. 2021. Metagenomic quantification of genes with internal standards. mBio 12:e03173-20. doi:10.1128/mBio.03173-20 PMC7858063 33531401

[54] Nayfach S, Pollard KS. 2016. Toward accurate and quantitative comparative metagenomics. Cell 166:1103–1116. doi:10.1016/j.cell.2016.08.007 27565341 PMC5080976

[55] Caverly LJ, Carmody LA, Haig SJ, Kotlarz N, Kalikin LM, Raskin L, LiPuma JJ. 2016. Culture-independent identification of nontuberculous mycobacteria in cystic fibrosis respiratory samples. PLoS ONE 11:e0153876. doi:10.1371/journal.pone.0153876 27093603 PMC4836755

[56] Johansen MD, Herrmann J-L, Kremer L. 2020. Non-tuberculous mycobacteria and the rise of Mycobacterium abscessus. Nat Rev Microbiol 18:392–407. doi:10.1038/s41579-020-0331-1 32086501

[57] Adjemian J, Olivier KN, Prevots DR. 2018. Epidemiology of pulmonary nontuberculous mycobacterial sputum positivity in patients with cystic fibrosis in the United States, 2010-2014. Ann Am Thorac Soc 15:817–826. doi:10.1513/AnnalsATS.201709-727OC 29897781 PMC6137684

[58] Donohue MJ. 2021. Epidemiological risk factors and the geographical distribution of eight Mycobacterium species. BMC Infect Dis 21:258. doi:10.1186/s12879-021-05925-y 33706712 PMC7953749

[59] Cazals M, Bédard E, Faucher SP, Prévost M. 2023. Factors affecting the dynamics of Legionella pneumophila, nontuberculous mycobacteria, and their host Vermamoeba vermiformis in premise plumbing. ACS EST Water 3:3874–3883. doi:10.1021/acsestwater.3c00288

[60] Delafont V, Mougari F, Cambau E, Joyeux M, Bouchon D, Héchard Y, Moulin L. 2014. First evidence of amoebae-mycobacteria association in drinking water network. Environ Sci Technol 48:11872–11882. doi:10.1021/es5036255 25247827

[61] Spencer-Williams I, Meyer M, DePas W, Elliott E, Haig S-J. 2023. Assessing the impacts of lead corrosion control on the microbial ecology and abundance of drinking-water-associated pathogens in a full-scale drinking water distribution system. Environ Sci Technol 57:20360–20369. doi:10.1021/acs.est.3c05272 37970641 PMC10702490

[62] Wang H, Masters S, Falkinham JO, Edwards MA, Pruden A. 2015. Distribution system water quality affects responses of opportunistic pathogen gene markers in household water heaters. Environ Sci Technol 49:8416–8424. doi:10.1021/acs.est.5b01538 26121595

[63] Gomez-Alvarez V, Ryu H, Tang M, McNeely M, Muhlen C, Urbanic M, Williams D, Lytle D, Boczek L. 2023. Assessing residential activity in a home plumbing system simulator: monitoring the occurrence and relationship of major opportunistic pathogens and phagocytic amoebas. Front Microbiol 14:1260460. doi:10.3389/fmicb.2023.1260460 37915853 PMC10616306

[64] Ringuet H, Akoua-Koffi C, Honore S, Varnerot A, Vincent V, Berche P, Gaillard JL, Pierre-Audigier C. 1999. hsp65 sequencing for identification of rapidly growing mycobacteria. J Clin Microbiol 37:852–857. doi:10.1128/JCM.37.3.852-857.1999 9986875 PMC84584

[65] Lee H, Park HJ, Cho SN, Bai GH, Kim SJ. 2000. Species identification of mycobacteria by PCR-restriction fragment length polymorphism of the rpoB gene. J Clin Microbiol 38:2966–2971. doi:10.1128/JCM.38.8.2966-2971.2000 10921960 PMC87161

[66] Adékambi T, Drancourt M. 2004. Dissection of phylogenetic relationships among 19 rapidly growing Mycobacterium species by 16S rRNA, hsp65, sodA, recA and rpoB gene sequencing. Int J Syst Evol Microbiol 54:2095–2105. doi:10.1099/ijs.0.63094-0 15545441

[67] Adékambi T, Colson P, Drancourt M. 2003. rpoB-based identification of nonpigmented and late-pigmenting rapidly growing mycobacteria. J Clin Microbiol 41:5699–5708. doi:10.1128/JCM.41.12.5699-5708.2003 14662964 PMC308974

[68] Alcaide F, Amlerová J, Bou G, Ceyssens PJ, Coll P, Corcoran D, Fangous M-S, González-Álvarez I, Gorton R, Greub G, et al.. 2018. How to: identify non-tuberculous Mycobacterium species using MALDI-TOF mass spectrometry. Clinical Microbiology and Infection 24:599–603. doi:10.1016/j.cmi.2017.11.012 29174730

[69] Rodriguez-Temporal D, Rodríguez-Sánchez B, Alcaide F. 2020. Evaluation of MALDI biotyper interpretation criteria for accurate identification of nontuberculous mycobacteria. J Clin Microbiol 58:e01103-20. doi:10.1128/JCM.01103-20 32719033 PMC7512167

[70] Rindi L, Puglisi V, Franconi I, Fais R, Lupetti A. 2022. Rapid and accurate identification of nontuberculous mycobacteria directly from positive primary MGIT cultures by MALDI-TOF MS. Microorganisms 10:1447. doi:10.3390/microorganisms10071447 35889166 PMC9317365

[71] El Khéchine A, Couderc C, Flaudrops C, Raoult D, Drancourt M. 2011. Matrix-assisted laser desorption/ionization time-of-flight mass spectrometry identification of mycobacteria in routine clinical practice. PLoS One 6:e24720. doi:10.1371/journal.pone.0024720 21935444 PMC3172293

[72] Rodriguez-Temporal D, Alcaide F, Mareković I, O’Connor JA, Gorton R, van Ingen J, Van den Bossche A, Héry-Arnaud G, Beauruelle C, Orth-Höller D, et al.. 2022. Multicentre study on the reproducibility of MALDI-TOF MS for nontuberculous mycobacteria identification. Sci Rep 12:1237. doi:10.1038/s41598-022-05315-7 35075208 PMC8786948

[73] van der Wielen PWJJ, Heijnen L, van der Kooij D. 2013. Pyrosequence analysis of the hsp65 genes of nontuberculous mycobacterium communities in unchlorinated drinking water in the Netherlands. Appl Environ Microbiol 79:6160–6166. doi:10.1128/AEM.01591-13 23913420 PMC3811344

[74] Waak MB, LaPara TM, Hallé C, Hozalski RM. 2019. Nontuberculous mycobacteria in two drinking water distribution systems and the role of residual disinfection. Environ Sci Technol 53:8563–8573. doi:10.1021/acs.est.9b01945 31287948

[75] Hull NM, Holinger EP, Ross KA, Robertson CE, Harris JK, Stevens MJ, Pace NR. 2017. Longitudinal and source-to-tap New Orleans, LA, U.S.A. drinking water microbiology. Environ Sci Technol 51:4220–4229. doi:10.1021/acs.est.6b06064 28296394

[76] Genilloud O. 2015. Micromonospora, p 1–28. In Whitman WB, Rainey F, Kämpfer P, Trujillo M, Chun J, DeVos P, Hedlund B, Dedysh S (ed), Bergey’s Manual of Systematics of Archaea and Bacteria, 1st ed. Wiley.

[77] Logan NA, Vos PD. 2015. Bacillus, p 1–163. In Whitman WB, Rainey F, Kämpfer P, Trujillo M, Chun J, DeVos P, Hedlund B, Dedysh S (ed), Bergey’s Manual of Systematics of Archaea and Bacteria, 1st ed. Wiley.

[78] Logan NA, Vos PD. 2015. Brevibacillus, p. 1–22. In Whitman, WB, Rainey, F, Kämpfer, P, Trujillo, M, Chun, J, DeVos, P, Hedlund, B, Dedysh, S (eds.), Bergey’s Manual of Systematics of Archaea and Bacteria, 1st ed. Wiley.

[79] Priest FG. 2015. Paenibacillus, p 1–40. In Whitman WB, Rainey F, Kämpfer P, Trujillo M, Chun J, DeVos P, Hedlund B, Dedysh S (ed), Bergey’s Manual of Systematics of Archaea and Bacteria, 1st ed. Wiley.

[80] Grigg C, Jackson KA, Barter D, Czaja CA, Johnston H, Lynfield R, Vagnone PS, Tourdot L, Spina N, Dumyati G, Cassidy PM, Pierce R, Henkle E, Prevots DR, Salfinger M, Winthrop KL, Toney NC, Magill SS. 2023. Epidemiology of pulmonary and extrapulmonary nontuberculous mycobacteria infections at 4 US emerging infections program sites: a 6-month pilot. Clin Infect Dis 77:629–637. doi:10.1093/cid/ciad214 37083882 PMC10444004

[81] Spaulding AB, Lai YL, Zelazny AM, Olivier KN, Kadri SS, Prevots DR, Adjemian J. 2017. Geographic distribution of nontuberculous mycobacterial species identified among clinical isolates in the United States, 2009-2013. Ann Am Thorac Soc 14:1655–1661. doi:10.1513/AnnalsATS.201611-860OC 28817307 PMC5711271

[82] Liang J, Swanson CS, Wang L, He Q. 2021. Impact of building closures during the COVID-19 pandemic on Legionella infection risks. Am J Infect Control 49:1564–1566. doi:10.1016/j.ajic.2021.09.008 34537274 PMC8447490

[83] Huang J, Chen S, Ma X, Yu P, Zuo P, Shi B, Wang H, Alvarez PJJ. 2021. Opportunistic pathogens and their health risk in four full-scale drinking water treatment and distribution systems. Ecological Engineering 160:106134. doi:10.1016/j.ecoleng.2020.106134

[84] Pinto AJ, Xi C, Raskin L. 2012. Bacterial community structure in the drinking water microbiome is governed by filtration processes. Environ Sci Technol 46:8851–8859. doi:10.1021/es302042t 22793041

[85] Griffith DE, Aksamit T, Brown-Elliott BA, Catanzaro A, Daley C, Gordin F, Holland SM, Horsburgh R, Huitt G, Iademarco MF, Iseman M, Olivier K, Ruoss S, von Reyn CF, Wallace RJ, Winthrop K, ATS Mycobacterial Diseases Subcommittee, American Thoracic Society, Infectious Disease Society of America. 2007. An official ATS/IDSA statement: diagnosis, treatment, and prevention of nontuberculous mycobacterial diseases. Am J Respir Crit Care Med 175:367–416. doi:10.1164/rccm.200604-571ST 17277290

[86] Moore JE, Kruijshaar ME, Ormerod LP, Drobniewski F, Abubakar I. 2010. Increasing reports of non-tuberculous mycobacteria in England, Wales and Northern Ireland, 1995-2006. BMC Public Health 10:612. doi:10.1186/1471-2458-10-612 20950421 PMC2964631

[87] Jones MM, Winthrop KL, Nelson SD, Duvall SL, Patterson OV, Nechodom KE, Findley KE, Radonovich LJ, Samore MH, Fennelly KP. 2018. Epidemiology of nontuberculous mycobacterial infections in the U.S. veterans health administration. PLoS ONE 13:e0197976. doi:10.1371/journal.pone.0197976 29897938 PMC5999224

[88] Prevots DR, Marshall JE, Wagner D, Morimoto K. 2023. Global epidemiology of nontuberculous mycobacterial pulmonary disease. Clinics in Chest Medicine 44:675–721. doi:10.1016/j.ccm.2023.08.012 37890910 PMC10625169

[89] Engers DW, Swarup R, Morrin C, Blauw M, Selfridge M, Gonyon P, Stout JE, Malani AN. 2023. A bronchoscopy-associated pseudo-outbreak of Mycobacterium chelonae and Mycobacterium mucogenicum associated with contaminated ice machine water and ice. Infect Control Hosp Epidemiol 44:2056–2058. doi:10.1017/ice.2023.101 37272469

[90] Boodman C, Smith C, von Kuster K, Lagacé-Wiens P, Wuerz T. 2021. Mycobacterium mucogenicum bacteremia and nodular soft tissue infection in a person who uses tap water to inject drugs. Open Forum Infect Dis 8:ofaa580. doi:10.1093/ofid/ofaa580 33447641 PMC7793457

[91] Tagashira Y, Kozai Y, Yamasa H, Sakurada M, Kashiyama T, Honda H. 2015. A cluster of central line-associated bloodstream infections due to rapidly growing nontuberculous mycobacteria in patients with hematologic disorders at a Japanese tertiary care center: an outbreak investigation and review of the literature. Infect Control Hosp Epidemiol 36:76–80. doi:10.1017/ice.2014.14 25627764

[92] Livni G, Yaniv I, Samra Z, Kaufman L, Solter E, Ashkenazi S, Levy I. 2008. Outbreak of Mycobacterium mucogenicum bacteraemia due to contaminated water supply in a paediatric haematology-oncology department. J Hosp Infect 70:253–258. doi:10.1016/j.jhin.2008.07.016 18799238

[93] Shachor-Meyouhas Y, Geffen Y, Arad-Cohen N, Zaidman I, Ben-Barak A, Davidson S, Kassis I. 2014. Mycobacterium phocaicum bacteremia: an emerging infection in pediatric hematology-oncology patients. Pediatr Infect Dis J 33:1299–1301. doi:10.1097/INF.0000000000000477 25037036

[94] Cooksey RC, Jhung MA, Yakrus MA, Butler WR, Adékambi T, Morlock GP, Williams M, Shams AM, Jensen BJ, Morey RE, Charles N, Toney SR, Jost KC, Dunbar DF, Bennett V, Kuan M, Srinivasan A. 2008. Multiphasic approach reveals genetic diversity of environmental and patient isolates of Mycobacterium mucogenicum and Mycobacterium phocaicum associated with an outbreak of bacteremias at a Texas hospital. Appl Environ Microbiol 74:2480–2487. doi:10.1128/AEM.02476-07 18310417 PMC2293142

[95] Kline S, Cameron S, Streifel A, Yakrus MA, Kairis F, Peacock K, Besser J, Cooksey RC. 2004. An outbreak of bacteremias associated with Mycobacterium mucogenicum in a hospital water supply. Infect Control Hosp Epidemiol 25:1042–1049. doi:10.1086/502341 15636290

[96] Alexander KJ, Furlong JL, Baron JL, Rihs JD, Stephenson D, Perry JD, Stout JE. 2021. Evaluation of a new culture medium for isolation of nontuberculous mycobacteria from environmental water samples. PLoS ONE 16:e0247166. doi:10.1371/journal.pone.0247166 33657154 PMC7928522

[97] Ditommaso S, Giacomuzzi M, Memoli G, Garlasco J, Curtoni A, Iannaccone M, Zotti CM. 2022. Chemical susceptibility testing of non-tuberculous mycobacterium strains and other aquatic bacteria: results of a study for the development of a more sensitive and simple method for the detection of NTM in environmental samples. J Microbiol Methods 193:106405. doi:10.1016/j.mimet.2021.106405 34990646

[98] Marshall JE, Gebert MJ, Lipner EM, Salfinger M, Falkinham III JO, Prevots DR, Mercaldo RA. 2023. Methods of isolation and identification of nontuberculous mycobacteria from environmental samples: a scoping review. Tuberculosis 138:102291. doi:10.1016/j.tube.2022.102291 36521261

[99] van der Wielen PWJJ, van der Kooij D. 2013. Nontuberculous mycobacteria, fungi, and opportunistic pathogens in unchlorinated drinking water in the Netherlands. Appl Environ Microbiol 79:825–834. doi:10.1128/AEM.02748-12 23160134 PMC3568566

[100] Rossman L, Woo H, Tryby M, Shang F, Janke R, Haxton T. 2020. EPANET (2.2.0). U.S. Environmental Protection Agency. Washington, DC, USA

[101] U.S. Environmental Protection Agency. 1997. Method 300.1: Determination of inorganic anions in drinking water by ion chromatography revision 1.0. U.S. Environmental Protection Agency, Cincinnati, Ohio. Available from: https://www.epa.gov/sites/default/files/2015-06/documents/epa-300.1.pdf

[102] Vosloo S, Sevillano M, Pinto A. 2019. Modified DNeasy PowerWater Kit protocol for DNA extractions from drinking water samples v1. doi:10.17504/protocols.io.66khhcw

[103] Standard Methods Committee of the American Public Health Association, American Water Works Association, and Water Environment Federation. 2017. Heterotrophic Plate Count. In Lipps WC, Baxter TE, Braun-Howland E (ed), Standard Methods For the Examination of Water and Wastewater, 23rd ed. APHA Press, Washington, D.C.

[104] Bustin SA, Benes V, Garson JA, Hellemans J, Huggett J, Kubista M, Mueller R, Nolan T, Pfaffl MW, Shipley GL, Vandesompele J, Wittwer CT. 2009. The MIQE guidelines: Minimum information for publication of quantitative real-time PCR experiments. Clin Chem 55:611–622. doi:10.1373/clinchem.2008.112797 19246619

[105] Borchardt MA, Boehm AB, Salit M, Spencer SK, Wigginton KR, Noble RT. 2021. The environmental microbiology minimum information (EMMI) guidelines: qPCR and dPCR quality and reporting for environmental microbiology. Environ Sci Technol 55:10210–10223. doi:10.1021/acs.est.1c01767 34286966

[106] Vosloo S, Huo L, Anderson CL, Dai Z, Sevillano M, Pinto A. 2021. Evaluating de novo assembly and binning strategies for time series drinking water metagenomes. Microbiol Spectr 9:e0143421. doi:10.1128/Spectrum.01434-21 34730411 PMC8567270

[107] Chen S, Zhou Y, Chen Y, Gu J. 2018. Fastp: an ultra-fast all-in-one FASTQ preprocessor. Bioinformatics 34:i884–i890. doi:10.1093/bioinformatics/bty560 30423086 PMC6129281

[108] Nurk S, Meleshko D, Korobeynikov A, Pevzner PA. 2017. metaSPAdes: a new versatile metagenomic assembler. Genome Res 27:824–834. doi:10.1101/gr.213959.116 28298430 PMC5411777

[109] Bushnell B. 2018. BBTools: a suite of fast, multithreaded bioinformatics tools designed for analysis of DNA and RNA sequence data. Joint Genome Institute.

[110] Gurevich A, Saveliev V, Vyahhi N, Tesler G. 2013. QUAST: quality assessment tool for genome assemblies. Bioinformatics 29:1072–1075. doi:10.1093/bioinformatics/btt086 23422339 PMC3624806

[111] Eren AM, Esen ÖC, Quince C, Vineis JH, Morrison HG, Sogin ML, Delmont TO. 2015. Anvi’o: an advanced analysis and visualization platform for ’omics data. PeerJ 3:e1319. doi:10.7717/peerj.1319 26500826 PMC4614810

[112] Alneberg J, Bjarnason BS, de Bruijn I, Schirmer M, Quick J, Ijaz UZ, Lahti L, Loman NJ, Andersson AF, Quince C. 2014. Binning metagenomic contigs by coverage and composition. Nat Methods 11:1144–1146. doi:10.1038/nmeth.3103 25218180

[113] Kang D, Li F, Kirton ES, Thomas A, Egan RS, An H, Wang Z. 2019. MetaBAT 2: an adaptive binning algorithm for robust and efficient genome reconstruction from metagenome assemblies. PeerJ. doi:10.7287/peerj.preprints.27522v1 PMC6662567 31388474

[114] Wu Y-W, Simmons BA, Singer SW. 2016. MaxBin 2.0: an automated binning algorithm to recover genomes from multiple metagenomic datasets. Bioinformatics 32:605–607. doi:10.1093/bioinformatics/btv638 26515820

[115] Sieber CMK, Probst AJ, Sharrar A, Thomas BC, Hess M, Tringe SG, Banfield JF. 2018. Recovery of genomes from metagenomes via a dereplication, aggregation and scoring strategy. Nat Microbiol 3:836–843. doi:10.1038/s41564-018-0171-1 29807988 PMC6786971

[116] Parks DH, Imelfort M, Skennerton CT, Hugenholtz P, Tyson GW. 2015. CheckM: assessing the quality of microbial genomes recovered from isolates, single cells, and metagenomes. Genome Res 25:1043–1055. doi:10.1101/gr.186072.114 25977477 PMC4484387

[117] Olm MR, Brown CT, Brooks B, Banfield JF. 2017. dRep: a tool for fast and accurate genomic comparisons that enables improved genome recovery from metagenomes through de-replication. ISME J 11:2864–2868. doi:10.1038/ismej.2017.126 28742071 PMC5702732

[118] Chaumeil P-A, Mussig AJ, Hugenholtz P, Parks DH. 2019. GTDB-Tk: a toolkit to classify genomes with the genome taxonomy database. Bioinformatics 36:1925–1927. doi:10.1093/bioinformatics/btz848 31730192 PMC7703759

[119] Aroney STN, Newell RJP, Nissen J, Camargo AP, Tyson GW, Woodcroft BJ. 2024. CoverM: read coverage calculator for metagenomics (0.7.0) 10.1093/bioinformatics/btaf147 PMC11993303 40193404

[120] R Core Team. 2021. R: a language and environment for statistical computing. R Foundation for Statistical Computing, Vienna, Austria.

[121] RStudio Team. 2020. RStudio: integrated development for R. RStudio. Boston, MA.

