# Supplementary material for: Source-to-tap investigation of the occurrence of nontuberculous mycobacteria in a full-scale chloraminated drinking water system
Source: Appl Environ Microbiol. 2024 Aug 7;90(9):e00609-24. doi: 10.1128/aem.00609-24 (PMC11409651; doi:10.1128/aem.00609-24)
Supplement: Supplemental material — Figures S1 to S10; Tables S1 to S6. [file aem.00609-24-s0001.pdf]

Supplementary Information

Source-to-Tap Investigation of the Occurrence of Nontuberculous Mycobacteria in a Full-Scale Chloraminated Drinking Water System

Authors

Katherine S. Dowdell,[1] Sarah C. Potgieter,[1] Kirk Olsen,[1] Soojung Lee,[1] Matthew Vedrin,[1] Lindsay J. Caverly,[2] John J. LiPuma,[2] and Lutgarde Raskin[*,1]

Author Affiliations

[1]Department of Civil and Environmental Engineering, University of Michigan, 1353 Beal Ave., Ann Arbor, MI 48109, USA

[2]Department of Pediatrics, University of Michigan Medical School, 8323 MSRB III, SPC5646, 1150 W. Med Cntr Dr., Ann Arbor, MI 48109, USA

[*]Corresponding Author

Lutgarde Raskin (raskin@umich.edu)

Contents

1. Supplementary Figures
2. Supplementary Tables

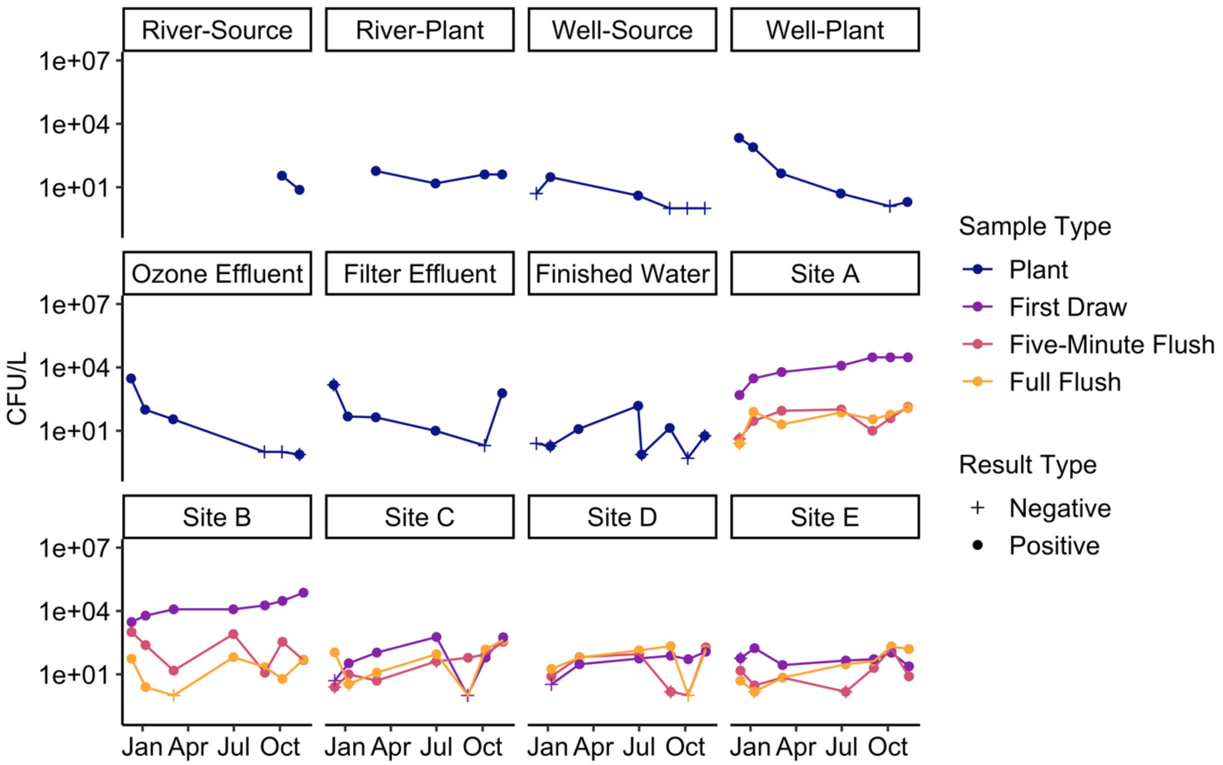

Figure S1. Presumptive average nontuberculous mycobacteria (NTM) plate culture results in colony forming units per liter (CFU/L) for all samples analyzed over the study period. Negative plates were set to one half the limit of detection (LOD) for plotting. Plates too numerous to count were set to 300 colonies for calculating averages and plotting. Source: Based on data from Dowdell et al., 2022 (1).

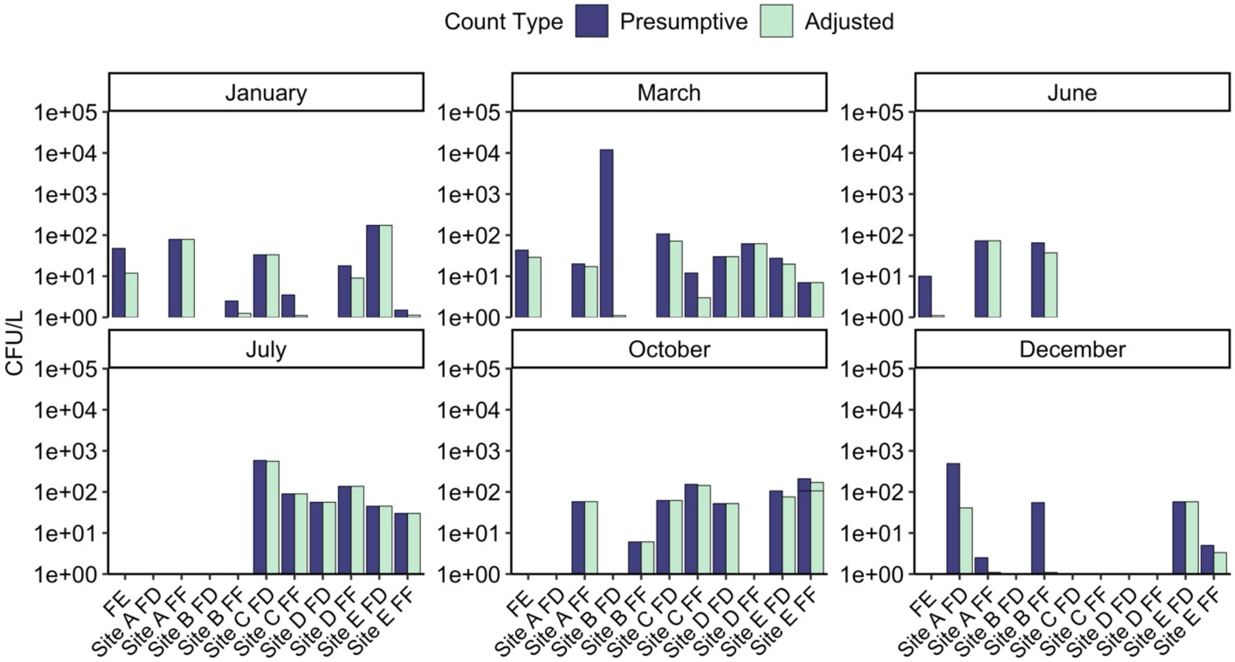

Figure S2. Presumptive and adjusted NTM plate counts in colony forming units per liter (CFU/L) of the filter effluent, building, and distribution system sample isolates. FE: filter effluent. Sites A-E: sampling sites in the distribution system, organized by estimated water age, from lowest water age (Site A) to highest water age (Site E). FD: first draw; FF: full flush. The presumptive NTM plate counts were adjusted based on the results of matrix-assisted laser desorption ionization-time of flight mass spectrometry (MALDI-TOF MS) analysis.

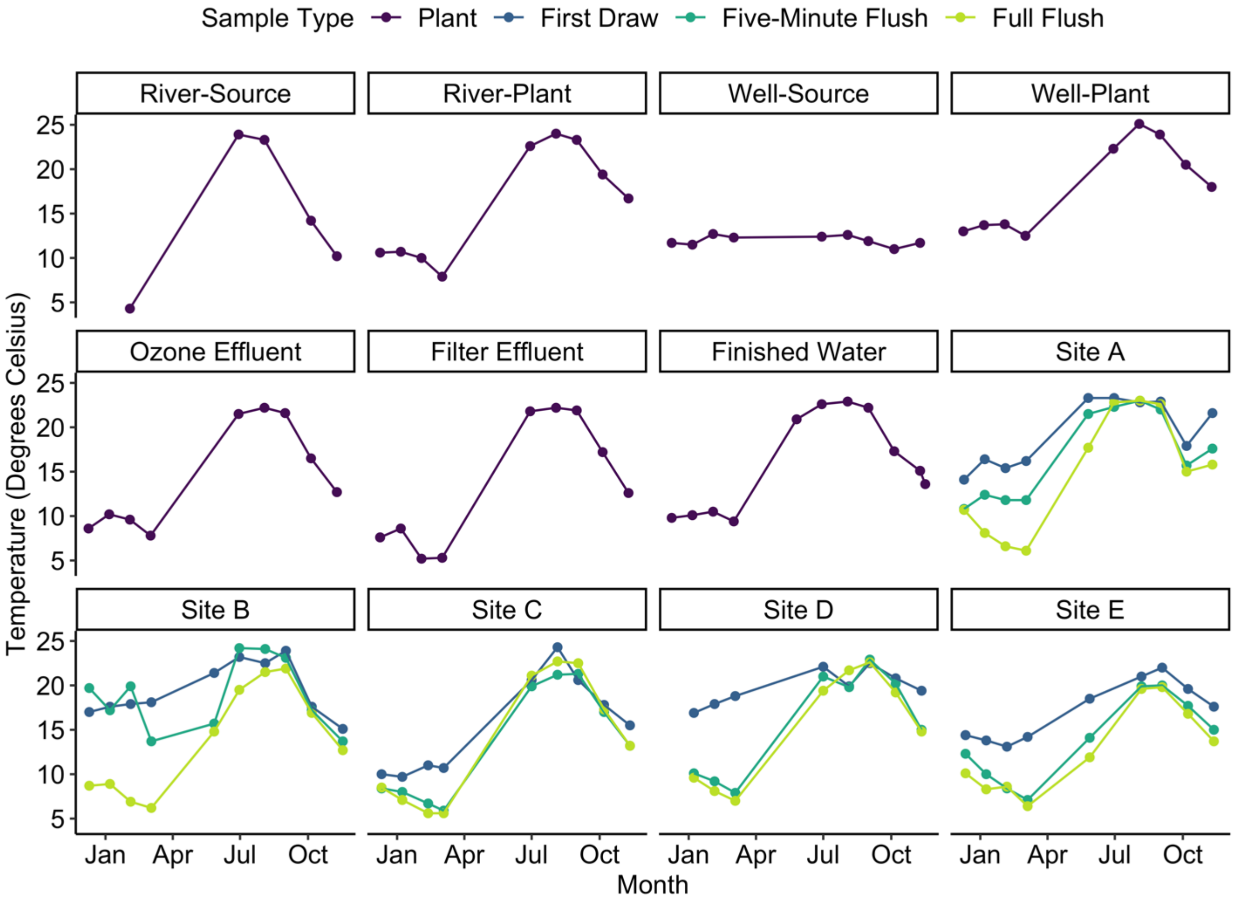

Figure S3. Sample temperature results (degrees Celsius) over the study period. Source: Based on data from Dowdell et al., 2022 (1).

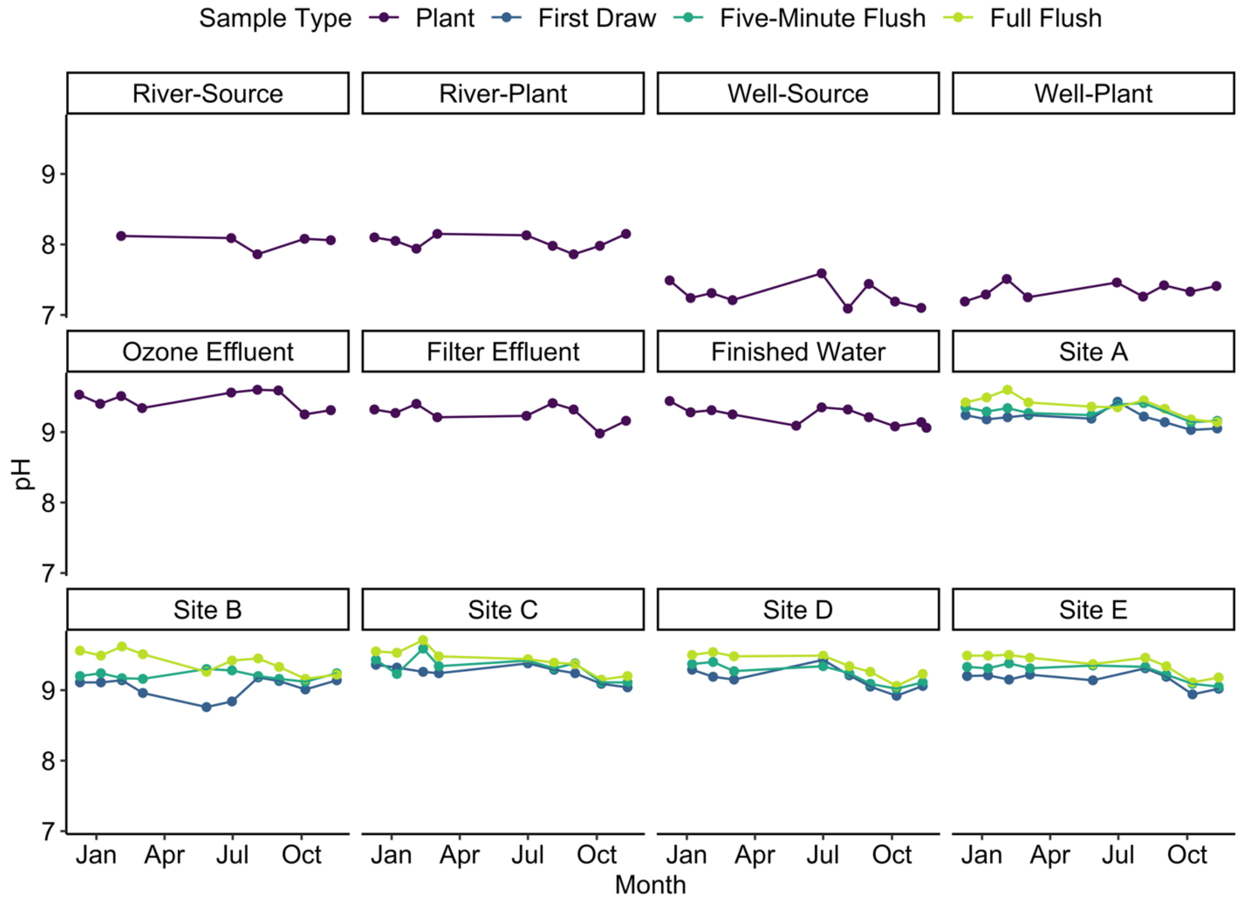

Figure S4. Sample pH results over the study period.

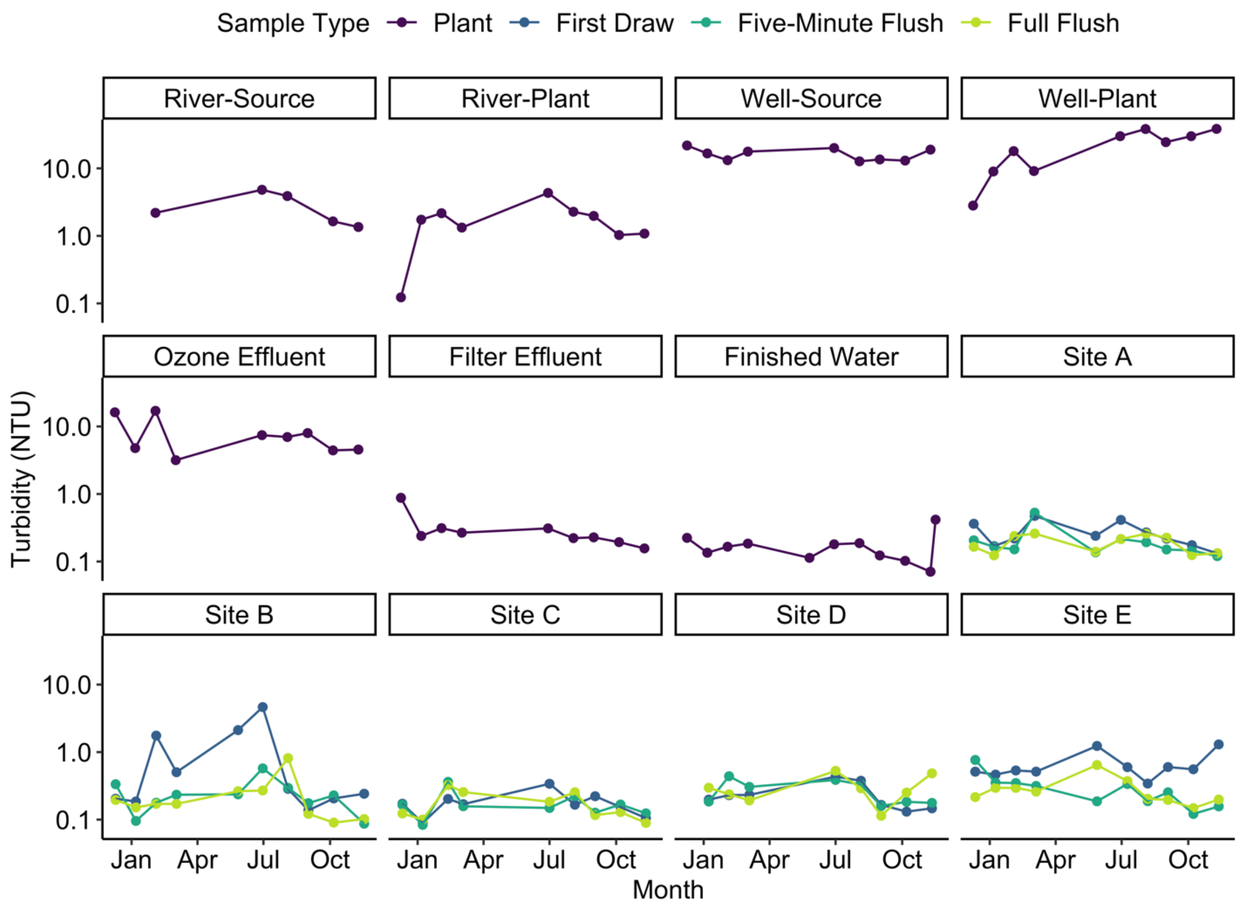

Figure S5. Sample turbidity (NTU) results over the study period.

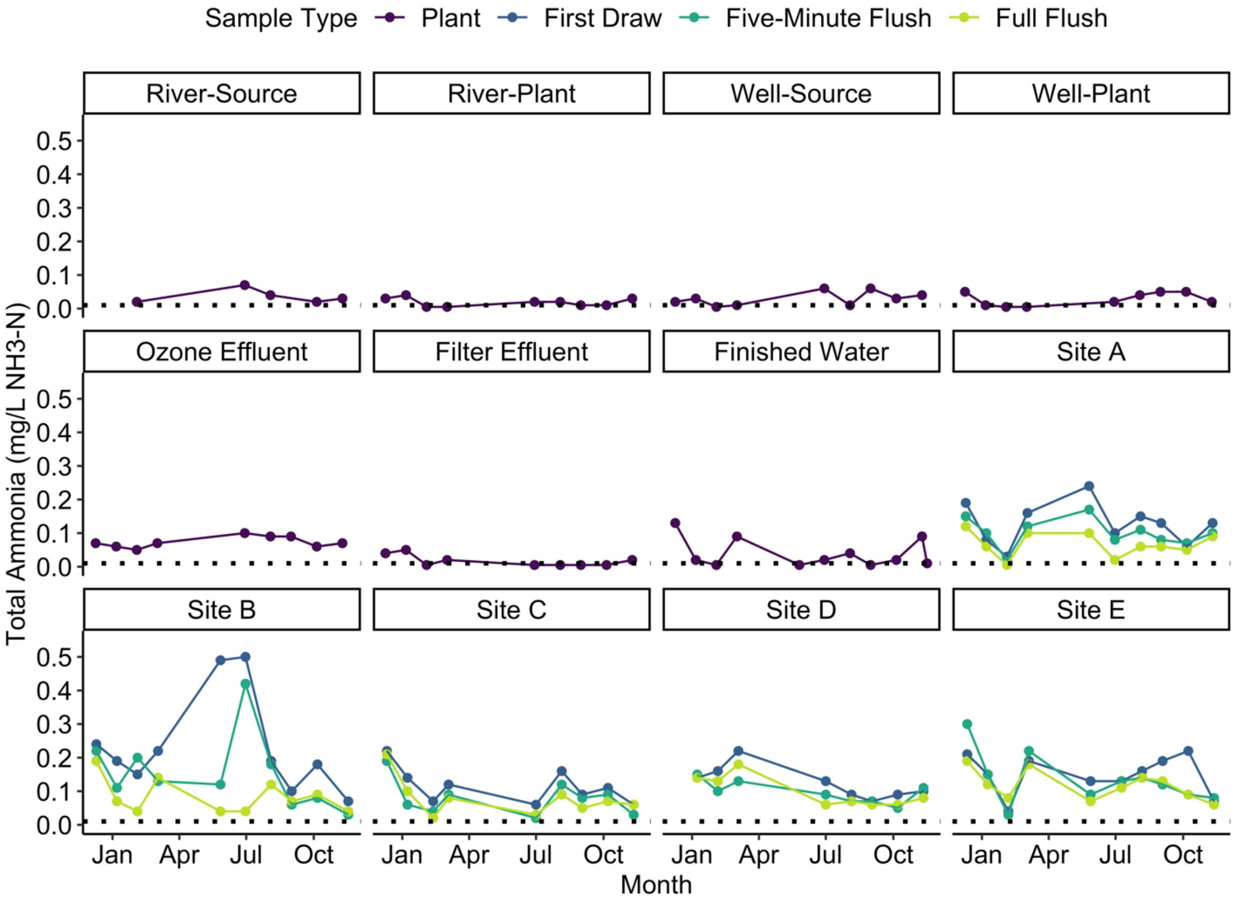

Figure S6. Total ammonia concentrations (mg/L NH_3-N) over the study period. Samples at the distribution system sites (Sites A – E) included first draw, five-minute flush, and full flush. The dotted line indicates the limit of detection (LOD, 0.01 mg/L NH_3-N).

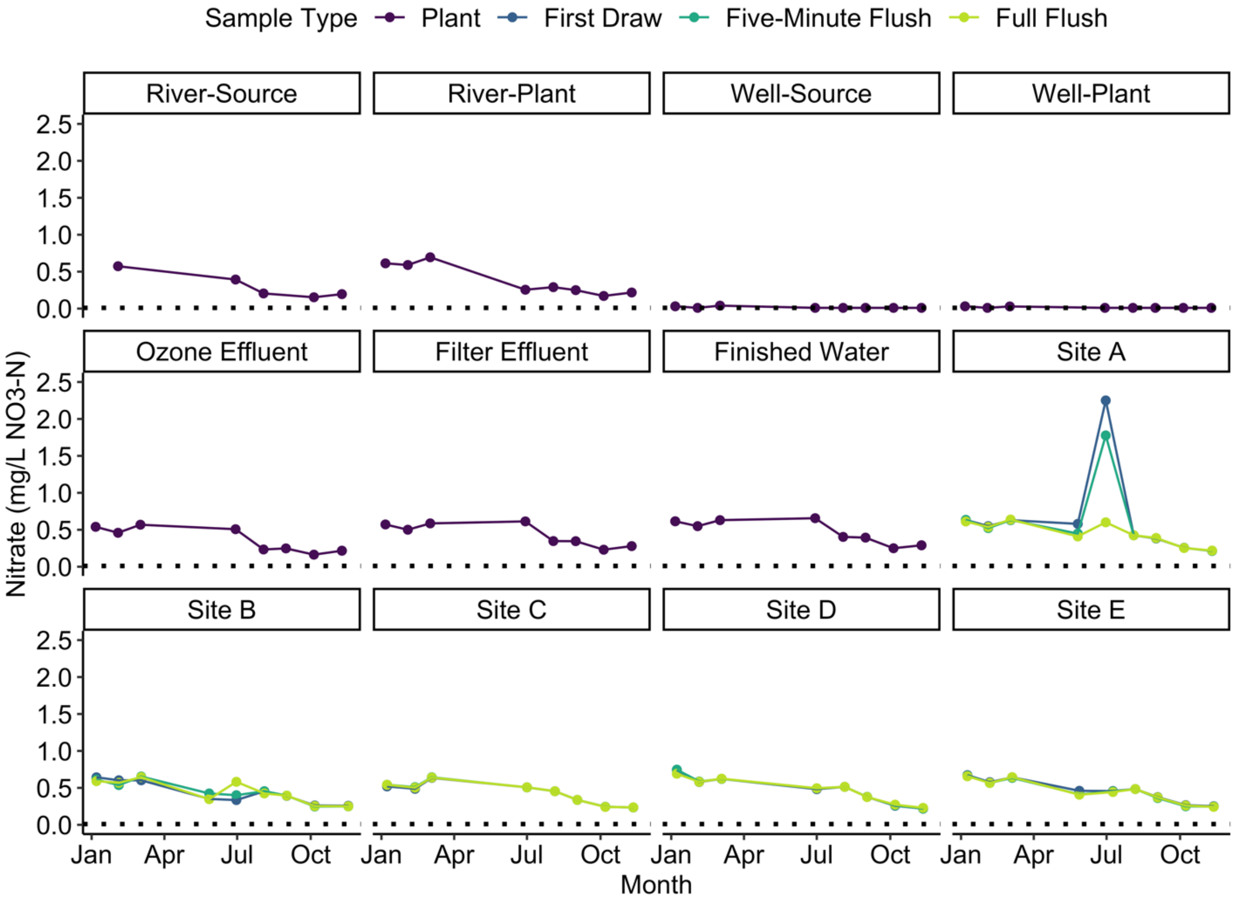

Figure S7. Nitrate concentrations (mg/L NO_3-N) over the study period. Samples at the distribution system sites (Sites A – E) included first draw, five-minute flush, and full flush. The dotted line indicates the limit of detection (LOD, 0.02 mg/L NO_3-N).

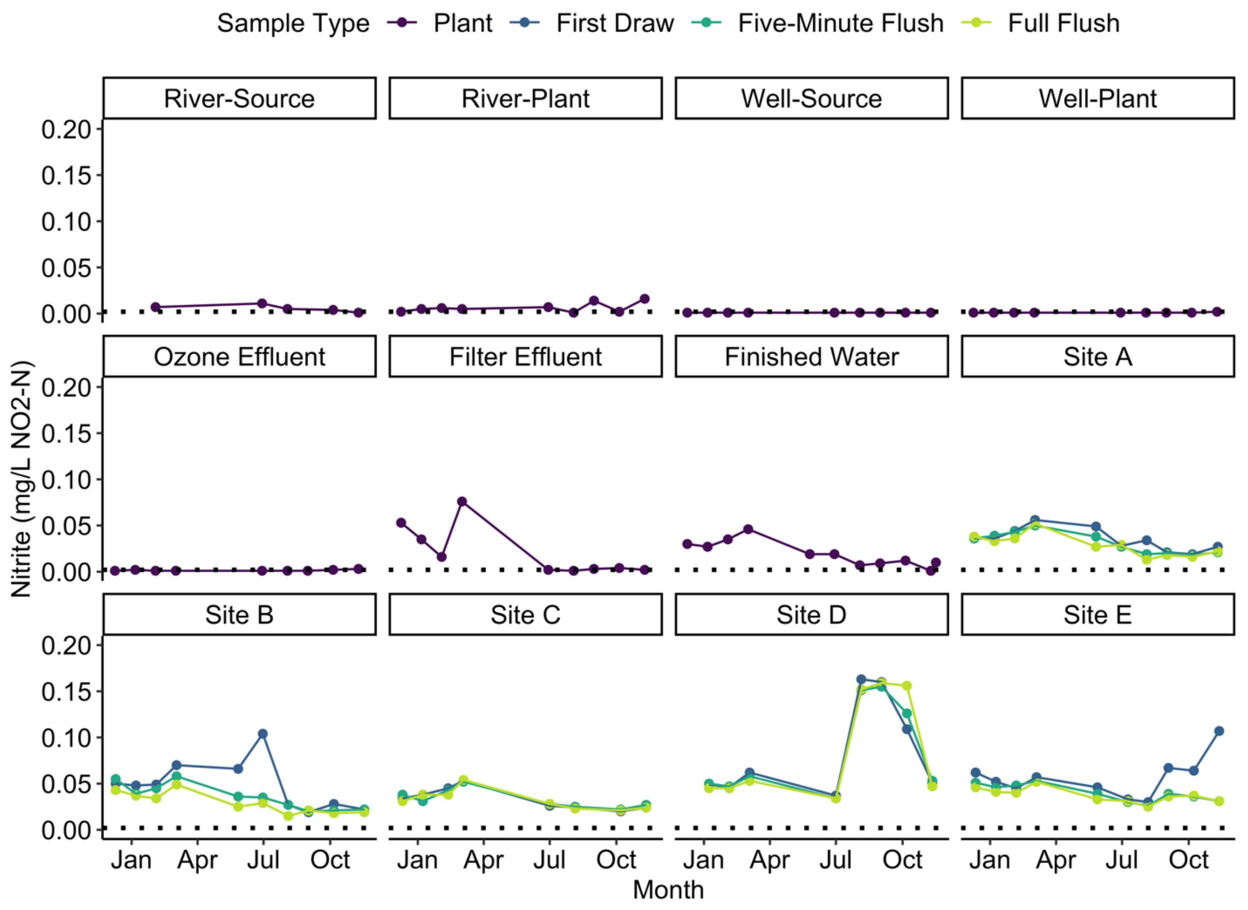

Figure S8. Nitrite concentrations (mg/L NO_2-N) over the study period. Samples at the distribution system sites (Sites A – E) included first draw, five-minute flush, and full flush. The dotted line indicates the limit of detection (LOD, 0.002 mg/L NO_2-N).

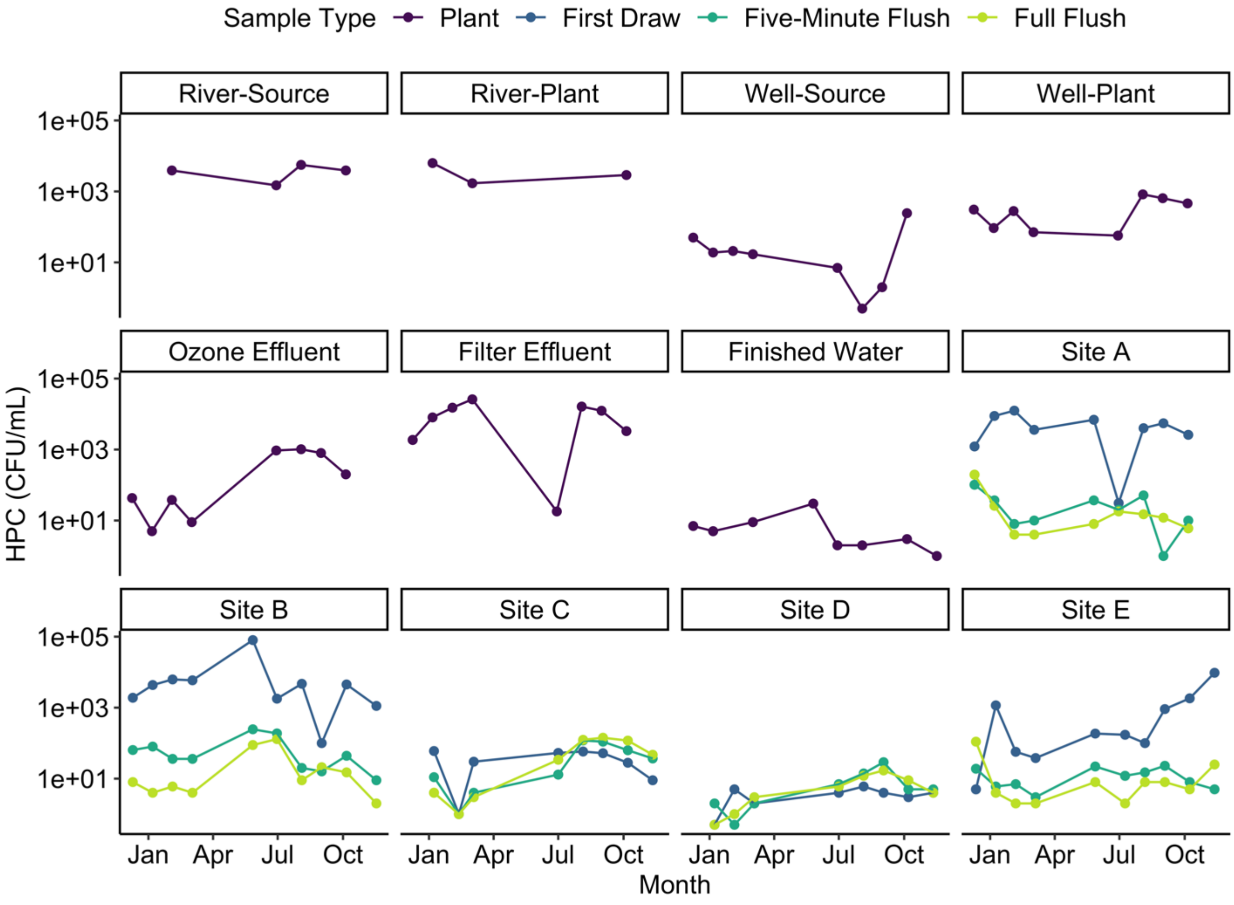

Figure S9. Sample heterotrophic plate count (HPC, CFU/mL) results over the study period.

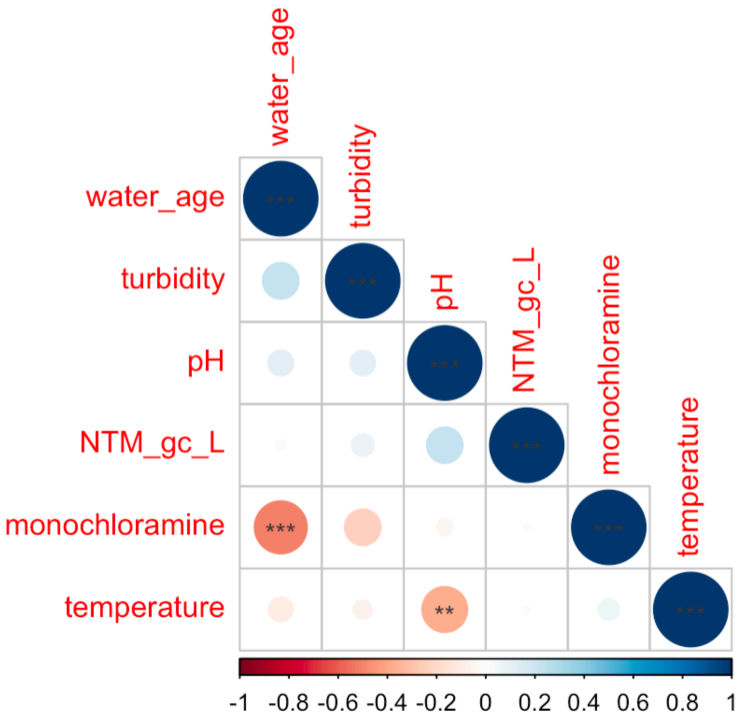

Figure S10. Correlation matrix for full flush distribution system samples (n=32) for total NTM using qPCR results (gene copies per liter, gc/L), water age, and physicochemical parameters using Kendall's tau b. The size and colors of the circles correspond to the bar below the correlation matrix, which indicates the strength of the association (Kendall's tau). An empty square indicates a very weak association (-0.02<Kendall's tau<0.02). The asterisks inside the circles show the significance (p-value). The absence of stars indicates that the association was not significant (p>0.05). *: p<0.05, **: p<0.01, ***: p<0.001.

2. Supplementary Tables

Table S1. Distribution system sampling location descriptions

Site	Building Type	Estimated Water Age (hours)[1]	Approx. Building Size	# Floors	Building Age (years)	Fixture Location & Description	Flow Rate (L/min)	Median Full Flush Time (min)
A	Municipal	16	78,000 ft^2 (7,246 m^2)	5	11 (2010)	Janitorial closet tap	17.1	21.5
B	Private	27	30,500 ft^2 (2,834 m^2)	2	43 (~1977)	Bathroom sink	7.5	23.0
C	Commercial	47	25,000 ft^2 (2,323 m^2)	1	6 (~2015)	Cold room dish washing sink	16.4	17.0
D	Commercial	56	47,000 ft^2 (4,366 m^2)	1	21 (~2000)	Handwashing Sink	7.5	17.5
E	Private	68	19,700 ft^2 (1,830 m^2)	2	45 (~1975)	Handwashing Sink	7.5	24.0

[1]Estimated using the City of Ann Arbor, Michigan's EPANET-based hydraulic model.

Table S2. Presumptive NTM plate culture results summary

Site	Type[1]	Colony Forming Units per Liter (CFU/L) by Sample[2]			Number of Plates[3]	Number of Samples[4]
		**Minimum**	**Median[5]**	**Maximum**		
River-Source	Bulk	7.5	21	35	4	2
River-Plant	Bulk	15	40	59	8	4
Well-Source	Bulk	<2	2.5	30	12	6
Well-Plant	Bulk	2	25	2,150	12	6
Ozone Effluent	Bulk	<1	18	>3,000	9	6
Filter Effluent	Bulk	<4	45	1503	12	6
Finished Water	Bulk	<1	4.1	152	15	8
Site A	FD	490	>12,000	>30,000	13	7
Site A	5M	4.2	39	135	14	7
Site A	FF	<3.3	58	119	14	7
Site B	FD	>3,000	>12,000	73,333	15	7
Site B	5M	12	243	>1,000	14	7
Site B	FF	<2	22	65	14	7
Site C	FD	<2	62	585	11	7
Site C	5M	<3.3	41.5	349	14	7
Site C	FF	<2	90	421	14	7
Site D	FD	<6.6	54	118	7	6
Site D	5M	<2	36.5	190	12	6
Site D	FF	<2	100	217	12	6
Site E	FD	24	52	173	10	7
Site E	5M	<2	8	168	14	7
Site E	FF	<2	30	209	14	7

[1]FD: first draw; 5M: five-minute flush; FF: full flush

[2]Plate counts are the average of duplicate plates unless there was insufficient volume for a duplicate or one of the plates could not be counted due to spreader colonies or contamination. The lower limit of detection (LLOD) was set at 1 CFU per volume processed and upper limit of detection (ULOD) was set at 300 CFU per volume. Average plate count results for each sample that were less than the LLOD were set at one-half the LLOD and results greater than the ULOD were set at the ULOD. Results that were below the LLOD or above the ULOD are reported as "< LLOD" or ">ULOD".

[3]Number of plates excludes plates considered "no count" due to spreader colonies or contamination.

[4]Number of samples excludes samples which yielded only "no count" plates.

[5]For even numbers of samples, the median is the average between the two middle values.

Table S3. Sample volumes used for NTM plate culture

Site	Type[1]	Plate Culture Volumes (mL)			Number of Plates[2]	Number of Samples
		Minimum	Median[3]	Maximum		
River-Source	Bulk	200	400	500	6	3
River-Plant	Bulk	100	200	500	14	7
Well-Source	Bulk	100	500	500	14	7
Well-Plant	Bulk	50	200	500	14	7
Ozone Effluent	Bulk	50	200	1000	14	7
Filter Effluent	Bulk	100	300	500	14	7
Finished Water	Bulk	200	1000	1000	16	8
Site A	FD	10	25	100	13	7
Site A	5M	300	500	500	14	7
Site A	FF	300	500	500	14	7
Site B	FD	3	25	100	15[4]	7
Site B	5M	200	300	500	14	7
Site B	FF	300	500	500	14	7
Site C	FD	100	200	500	11	7
Site C	5M	300	500	500	14	7
Site C	FF	300	500	500	14	7
Site D	FD	150	500	500	7	6
Site D	5M	481	500	500	12	6
Site D	FF	500	500	500	12	6
Site E	FD	100	200	500	10	7
Site E	5M	300	500	500	14	7
Site E	FF	300	500	500	14	7

[1]FD: first draw; 5M: five-minute flush; FF: full flush

[2]Includes all plates, including those that count not be counted (“no count”, “too numerous to count”, countable, and negative plates).

[3]For even numbers of plates, the median is the average between the two middle values.

[4]Includes an additional plate from a duplicate sample.

Table S4. Results of MALDI-TOF MS analysis of isolates from NTM culture plates by genus and species

Genus	Microorganism	MALDI-TOF Scores[1]			Count
		Minimum	Median[2]	Maximum	
Bacillus	*Bacillus cereus*	1.73	1.91	2.04	5
	Bacillus megaterium	1.94	1.94	1.94	1
	Bacillus pumilus	1.70	1.70	1.70	1
	Bacillus safensis	1.81	1.81	1.81	1
	Bacillus thermoamylovorans	2.15	2.25	2.34	2
	Bacillus thuringiensis	1.95	1.95	1.95	1
Brevibacillus	*Brevibacillus borstelensis*	1.77	1.96	2.40	6
Micromonospora	*Micromonospora aurantiaca*	1.73	1.73	1.73	1
Mycobacterium	*Mycobacterium arupense*	1.92	1.94	1.96	2
	Mycobacterium asiaticum	1.61	1.61	1.61	1
	Mycobacterium aurum	1.98	2.13	2.27	4
	Mycobacterium avium	1.99	2.09	2.20	8
	Mycobacterium chelonae complex	1.62	1.88	2.12	84
	Mycobacterium franklinii	1.60	1.77	1.98	19
	Mycobacterium gordonae	1.81	1.91	2.01	2
	Mycobacterium llatzerense	1.84	1.84	1.84	1
	Mycobacterium mucogenicum/phocaicum group	1.82	2.11	2.39	65
	Mycobacterium peregrinum	2.10	2.27	2.31	8
Paenibacillus	*Paenibacillus azoreducens*	2.00	2.00	2.00	1
	Paenibacillus polymyxa	1.82	1.82	1.82	1
None	No identification	0.93	1.21	1.68	108

[1]Per manufacturer guidelines, the minimum score for nontuberculous mycobacteria (NTM) identification was 1.60 and the minimum score for non-NTM was 1.70.

[2]For even numbers of isolates, the median is the average between the two middle values.

Table S5. NTM qPCR results summary

Site	Type[1]	Number of Samples	Volume Filtered (L)			qPCR Result (gene copies per L)[2]		
			**Minimum**	**Median[3]**	**Maximum**	**Minimum**	**Median[3]**	**Maximum**
River-Source	Bulk	4	0.4	0.6	1.2	2.1x10^{4}	1.1x10^{5}	1.8x10^{5}
River-Plant	Bulk	7	0.4	1.1	1.3	8.3x10^{3}	6.8x10^{4}	5.3x10^{5}
Well-Source	Bulk	7	6.9	8.9	18.1	<2.3x10^{2}	<4.6x10^{2}	<6.0x10^{2}
Well-Plant	Bulk	7	8.5	9.0	16.6	<2.8x10^{2}	1.6x10^{3}	3.7x10^{4}
Ozone Effluent	Bulk	7	7.4	9.0	19.3	<2.2x10^{2}	<4.9x10^{2}	<4.3x10^{3}
Filter Effluent	Bulk	7	7.3	8.4	9.9	3.8x10^{3}	1.1x10^{4}	8.6x10^{4}
Finished Water	Bulk	8	7.8	10.0	11.7	<3.5x10^{2}	1.4x10^{3}	3.9x10^{4}
Site A	FD	8	0.9	1.0	1.2	2.3x10^{4}	1.3x10^{5}	5.5x10^{5}
Site A	5M	8	6.5	8.3	9.9	6.1x10^{2}	7.5x10^{3}	7.3x10^{4}
Site A	FF	8	6.8	11.5	13.7	<3.4x10^{2}	9.2x10^{3}	5.4x10^{4}
Site B	FD	8	0.7	1.1	1.8	4.1x10^{5}	7.7x10^{6}	4.2x10^{7}
Site B	5M	7	7.5	8.4	9.2	4.5 x10^{4}	3.0 x10^{5}	2.2x10^{6}
Site B	FF	7	8.0	9.6	11.8	1.4x10^{4}	1.0x10^{5}	3.2x10^{5}
Site C	FD	6	0.8	1.1	1.3	<3.2x10^{3}	1.8x10^{4}	3.3x10^{4}
Site C	5M	6	7.4	8.4	8.5	2.4x10^{3}	1.7x10^{4}	6.8x10^{4}
Site C	FF	6	8.9	11.3	12.2	8.4x10^{2}	7.1x10^{3}	2.7x10^{4}
Site D	FD	5	0.8	1.0	1.2	1.1x10^{4}	3.5x10^{4}	7.8x10^{4}
Site D	5M	6	8.3	8.6	10.6	7.8x10^{3}	3.0x10^{4}	7.1x10^{4}
Site D	FF	5	8.0	12.0	12.7	2.9x10^{3}	3.0x10^{4}	5.0x10^{4}
Site E	FD	7	0.9	1.1	1.5	<4.7x10^{3}	5.1x10^{4}	1.5x10^{5}
Site E	5M	7	7.5	8.3	8.9	9.4x10^{2}	4.2x10^{3}	1.8x10^{5}
Site E	FF	7	8.4	10.3	11.8	<9.7x10^{2}	5.3x10^{3}	1.8x10^{5}

[1]FD: first draw; 5M: five-minute flush; FF: full flush

[2]Results less than the limit of quantification (LOQ, 41 gene copies per reaction) are reported as "<LOQ" in gene copies per L.

[3]For even numbers of samples, the median is the average between the two middle values.

Table S6. Statistics and quality estimates of *Mycobacterium* metagenome-assembled genomes (MAGs). The completeness and redundancy of each MAG was determined using CheckM (v1.0.18).

Species	Completeness (%)	Redundancy (%)	Strain heterogeneity	Genome size (bp)	No. contigs	N50 contigs	Longest contig (bp)	GC content (%)
Mycobacterium sp.	68.77	2.9	68.42	4755905	6137	304	1580	66.58
Mycobacterium arupense	99.55	1.18	0	4869182	4704	381	3037	64.47
Mycobacterium phocaicum	56.74	1.64	60	4734168	5229	350	3207	66.73
Mycobacterium llatzerense	70.31	2.34	46.15	4771175	5085	357	5797	66.57
Mycobacterium gordonae	96.29	2.16	22.22	6564682	5967	399	6987	66.75

bp: base pair

References

1. Dowdell KS, Raskin L, Olson T, Haig S-J, Dai D, Edwards M, Pruden A. 2022. Methods for Detecting and Differentiating Opportunistic Premise Plumbing Pathogens (OPPPs) to Determine Efficacy of Control and Treatment Technologies. Report 4721. Water Research Foundation, Denver, CO.
